# A context-aware transformer with weighted residual for efficient remote sensing target detection

**DOI:** 10.3389/fnbot.2026.1774633

**Published:** 2026-05-15

**Authors:** Zhongyu Li, Yimin Shen, Xiaoping Jing, Rong Wang, Di Zhou, Yangyong Guo, Wei Yu, Fei Du

**Affiliations:** 1 Chengdu Technological University, Chengdu, China; 2 Key Laboratory of Interior Layout Optimization and Security, Institutions of Higher Education of Sichuan Province, Chengdu Normal University, Chengdu, China; 3 Intelligent Optoelectronic Perception System and Applications Key Laboratory of Sichuan Provincial Universities, Sichuan University of Arts and Science, Dazhou, China; 4 Artificial Intelligence Key Laboratory of Sichuan Province, Yibin, China; 5 Sichuan University of Arts and Science, Dazhou, China; 6 Chengdu University of Information Technology, Chengdu, China

**Keywords:** disaster remote sensing images, loss imbalance, rotated target, target detection, weighted residual pyramid

## Abstract

Remote sensing image target detection has important applications in disaster prevention and mitigation. However, current detection models still have shortcomings in multi-scale feature fusion and in representing contextual information, and are prone to class imbalance during training. To address these issues, this paper proposes a target detection model that combines a context transformer and a weighted residual pyramid. Specifically, a weighted residual pyramid module is designed to fuse deep and shallow target-instance features effectively, and a learnable balancing factor is introduced to alleviate the imbalance in contributions across network layers. Simultaneously, a context transformer module is introduced into the feature extraction and fusion networks to enhance the model’s multi-scale feature representation capability. Furthermore, a rotated bounding box is introduced to locate target instances, reducing the influence of redundant background information, and a CIoU-based multi-task loss function is designed to reduce the contribution of different instance targets to the regression task. Experimental results show that, while ensuring real-time performance, the proposed model achieves mAP@0.5 scores of 0.754 and 0.714 on the DOTAv1.0 and DOTAv1.5 datasets, respectively, demonstrating consistent improvements across both datasets. Meanwhile, visualization of detection results across various disaster scenarios shows that the model proposed in this paper has strong practical value.

## 1 Introduction

With the development of remote sensing technology, the storage volume of high-resolution remote sensing imagery is increasing daily, providing crucial data support for related research in Earth sciences (Gao et al., 2024). High-resolution remote sensing images can clearly present the geometry and spatial distribution of ground features, providing favorable conditions for high-quality remote sensing target interpretation (Fan et al., 2024). Urban planners need a clear understanding of land conditions, transportation, and other factors within their jurisdiction to support informed planning decisions; natural resource managers need to understand the distribution of wetlands, forests, rivers, and other natural features to provide a basis for ecological simulation. Therefore, high-resolution remote sensing imagery, with its unique advantages, is widely used in land resource surveys, ecological environment simulation, urban planning, and disaster prevention and mitigation. The efficient and comprehensive utilization of high-resolution remote sensing imagery is of great significance in the military, civilian, and ecological fields (Li et al., 2024). With the development of artificial intelligence technology, deep neural networks have been widely applied in fields such as pedestrian trajectory prediction (Lui et al., 2025) and remote sensing image processing (Li J. et al., 2025). Remote sensing image target detection requires identifying the locations of target instances and labeling their categories, which is fundamental to solving complex, abstract tasks such as scene segmentation and target tracking (Huang et al., 2025). Traditional manual feature extraction methods for target detection rely on features such as colour, texture, and shape, and require processes such as candidate region generation, feature extraction, and classification. Especially in feature extraction, they rely heavily on expert knowledge. Therefore, this method performs poorly in remote sensing images with complex backgrounds and severe interference. Convolutional deep neural networks, with their powerful feature-extraction capabilities, have replaced traditional manual feature extraction and have become the primary approach for remote sensing image target detection (Wu et al., 2025). Remote sensing image target detection models based on deep neural networks are mainly divided into single-stage and two-stage models. Single-stage models include YOLO (You Only Look Once), while two-stage models include R-CNN (Region-based Convolutional Neural Network). Two-stage target detection models typically require generating candidate bounding boxes using an algorithm, followed by neural network classification and regression. Typical two-stage models include R-CNN (Girshick et al., 2014), Fast-RCNN (Girshick, 2015), Faster-RCNN (Ren et al., 2016), Mask R-CNN (He et al., 2017), Cascade R-CNN (Cai and Vasconcelos, 2018), Sparse R-CNN (Sun et al., 2021), and Oriented R-CNN (Xie et al., 2024), etc. Although the aforementioned methods have made good progress in general target detection, they still face many challenges in remote sensing scenarios with complex backgrounds and large variations in target scale.

In remote sensing image target detection, target instances exhibit a multi-scale and dense distribution. Current mainstream target detection models lack effective collaboration between different network layers and the global context, resulting in insufficient multi-scale representation of instance features. Furthermore, in bounding box-based target detection models, targets of different sizes contribute differently to the regression loss. Simultaneously, the intersection-over-union (IoU) between the predicted and ground-truth bounding boxes varies by target. The range, gradient contribution, and training difficulty of the loss function also vary by task when constructing localization and regression subtasks. Moreover, the inconsistent contribution of features at different levels of the target instance to the loss leads to severe loss imbalance, posing a significant challenge to target detection in remote sensing images. To alleviate these problems, this paper designs a target detection model that integrates contextual information and multi-scale features. By introducing a context transformer module and designing a weighted residual pyramid structure, a more effective fusion of multi-scale features and contextual information is achieved, enhancing feature representation while effectively mitigating the loss imbalance problem. The main contributions are as follows:

(1) An improved rotated bounding box is used for target instance localization to reduce the impact of redundant background information.
(2) A context transformer module is introduced, combined with a weighted residual pyramid structure, to achieve collaborative fusion of deep and shallow features and contextual information in multi-scale target instances, thereby alleviating the imbalance of feature contributions at different levels.
(3) A multi-task weighted rotated bounding box loss function is designed to reduce the imbalance of loss contributions from different target instances to the regression task.
(4) The effectiveness of the model is verified using the DOTAv1.0 and DOTAv1.5 datasets.
(5) Tests were conducted in various disaster remote sensing image scenarios, achieving good detection results.

The remainder of this paper is organized as follows: Section 2 discusses related work; Section 3 discusses materials and methods; Section 4 discusses and analyzes experimental results; and Section 5 presents conclusions and future work.

## 2 Related works

### 2.1 Mainstream object detection methods

Currently, methods for remote sensing image target detection using deep neural networks primarily aim to alleviate class imbalance by redesigning the feature discriminator or by employing multi-scale feature fusion techniques. To address the high computational complexity of reconstructing the target detection discriminator, the literature (Tao et al., 2025) proposes an efficient single-stage detector (EDADet) for small targets. This model uses domain conversion technology to fuse multimodal data across domains from single-modal inputs. A backbone network for small-target perception is then designed to facilitate feature fusion and extraction in conjunction with the encoder-decoder feature fusion (EDFF) architecture. To address the problem of target detection models misclassifying small-class samples, the paper (An et al., 2025) proposed a detection network that leverages discriminative feature learning and imbalanced-feature semantic expansion modules. The model constructs clear classification boundaries and represents scarce-class features by introducing discriminative feature-learning modules and imbalanced-feature semantic-expansion modules. To address the problem of unbalanced background distributions in remote sensing images, the paper (Rao and Zhou, 2024) proposed a cross-grid label assignment (CLA) method that alleviates the imbalance between positive and negative samples by adding high-quality positive samples for training and loss computation and by designing a feature refinement head (FRHead). To address the multi-scale transformation problem in remote sensing image targets, Gao et al. (2024) proposed a new few-shot target detection model that improves support and query features by leveraging contextual information of multi-scale targets through a feature aggregation module (FAM) and a scale-aware attention module (SAM). To address gradient conflicts and class imbalance in small-target detection in remote sensing images, a two-stage balanced orthogonal subspace separation (BOSS) detector has been proposed (Jiang et al., 2024). The low-rank subspace adapter (LoSA) was introduced into the model for structural separation. At the same time, the orthogonal subspace extractor (OSE) and balanced classifier (BC) were designed for feature disentanglement and balanced imbalance loss, respectively. Reference (Pang et al., 2019) proposed the Libra R-CNN model to address the imbalance problem caused by varying contributions of different layers to the IoU, classification, and regression losses during training. This model employs an IoU-balanced sampling module to mitigate sample imbalance, a balanced pyramid module to address feature imbalance, and a balanced L1 loss to alleviate the imbalance between classification and regression tasks. Reference (Rezatofighi et al., 2019) proposed the GIoU (Generalized IoU) loss to address the issue that when the predicted box does not overlap with the instance target’s actual box, the IoU is 0. Reference (Zheng et al., 2020) proposed the DIoU (Distance IoU) loss to address the problem of the GIoU loss. This method considers both the overlap between bounding boxes and the distance to the center point, converging quickly; however, it does not account for differences in aspect ratio between the predicted and target bounding boxes. To address this problem, the research team of the DIoU loss paper proposed the CIoU (Complete IoU) loss in the same article. Reference (Zhang et al., 2022) proposed the EIoU (Efficient Intersection over Union) loss to address the limitation that the CIoU loss uses only a single factor to quantify the aspect-ratio difference between bounding boxes, which cannot accurately assess its magnitude and may hinder effective network optimization. This addresses the issue of an unclear definition of the bounding-box aspect-ratio and introduces Focal Loss to mitigate the imbalance between hard and easy samples. Reference (Cao et al., 2020) investigated the impact of different samples on the average accuracy of target detection across categories and proposed the PISA (Prime Sample Attention) method to enhance the model’s performance. Reference (Oksuz et al., 2021) addressed the problem of unequal task importance in multi-task and multi-stage neural network training and proposed the RS (Rank and Sort) Loss, which effectively simplified the model and improved performance. To address the scale imbalance problem in remote sensing image target detection, Li Z. et al. (2025) proposed a network based on a frequency-domain attention mechanism. It verified the model’s effectiveness in real-world remote-sensing imagery. To address the problem that CNNs cannot capture long-distance dependencies and the high computational cost of the attention mechanism, Liu et al. (2024) proposed a feature pyramid full-granular attention network (FPFGANet). The above method primarily enhances the model through its network structure. It does not fully account for the impact of target instances in remote sensing images on the target-detection task’s loss.

### 2.2 Bottlenecks and shortcomings of current research

Due to the unique imaging characteristics of remote sensing images, despite some progress in existing target detection methods, the following problems persist, especially given the complex backgrounds and large-scale variations in target instances. These problems also limit the feature representation capabilities of existing models:

(1) Target instances in remote sensing images possess multi-angle features. Traditional horizontal bounding box methods struggle to determine the orientation of target instances accurately and to avoid introducing non-instance information, thereby affecting the model’s learning of target-instance features.
(2) Target instances in remote sensing images contain significant background interference, which impacts the image detection model’s ability to identify target instances, particularly during multi-scale feature fusion.
(3) Remote sensing image target detection integrates multiple sub-tasks such as localization and classification. Different sub-tasks contribute differently to the loss function, leading to an imbalance during training and affecting the overall performance of the detection model.

Currently, with the continuous development of the YOLO series of object detection models, advanced versions such as YOLO10 have been developed. However, CSPDarknet53 (Jocher et al., 2021; Bochkovskiy et al., 2020) has a more stable structure, more mature code, and stronger compatibility. Considering both stability and compatibility, this paper uses CSPDarknet53 as the base network to improve the model’s adaptability in complex remote sensing images. This structure has a good foundation in multi-scale feature representation. Based on the above-mentioned basic structure and the analysis of challenges in remote sensing object detection tasks, and addressing the shortcomings of current detection models in multi-scale feature fusion and contextual information representation, which easily leads to loss imbalance during training, this paper constructs an object detection model that combines a context transformer and a weighted residual pyramid. By combining the context transformer and weighted residual pyramid modules, the ability to represent multi-scale features and contextual information is increased, alleviating the loss imbalance problem. The performance of this model was validated on the DOTAv1.0 and DOTAv1.5 datasets, achieving strong detection performance across diverse remote-sensing scenarios of random disasters.

## 3 Materials and methods

### 3.1 Overall framework

**Figure 1:**
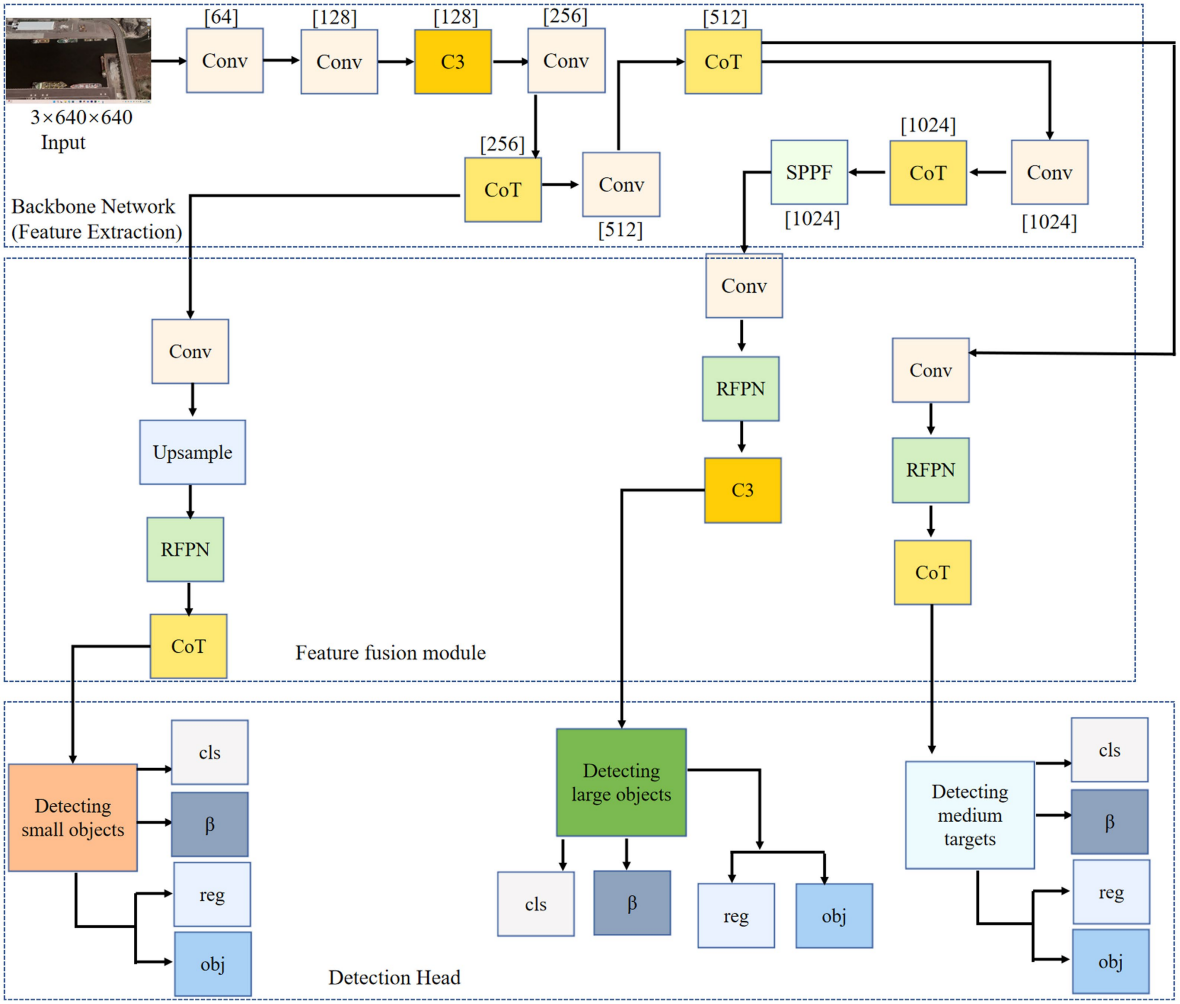
Overall framework of the proposed method: the input remote sensing image is processed by the backbone network to extract features. The detection head predicts bounding boxes and classification scores, and the proposed loss module mitigates the imbalance in IoU loss.

Figure 1 illustrates the overall structure of the proposed model, which consists of three parts: a feature-extraction backbone network, a feature-fusion network, and a detection head. CoT modules are integrated into the backbone and feature extraction networks to enhance the model’s feature representation by establishing spatial and channel-wise contextual relationships, thereby improving the ability to distinguish target and background regions and to represent features in complex backgrounds. In the feature fusion network, a residual feature pyramid module is designed to enhance information interaction between features at different scales, thereby addressing insufficient multi-scale feature fusion. Simultaneously, learnable weight factors are introduced to adjust the fusion contribution of features at different scales. In the detection head, rotated bounding boxes are used for target instance localization to adapt to the arbitrary directional distribution of targets in remote sensing images, reducing redundant background interference and improving localization accuracy. A weighted loss function based on rotated bounding boxes is designed to alleviate the imbalance of losses among different target instances in the regression task.

### 3.2 Backbone

**Figure 2:**
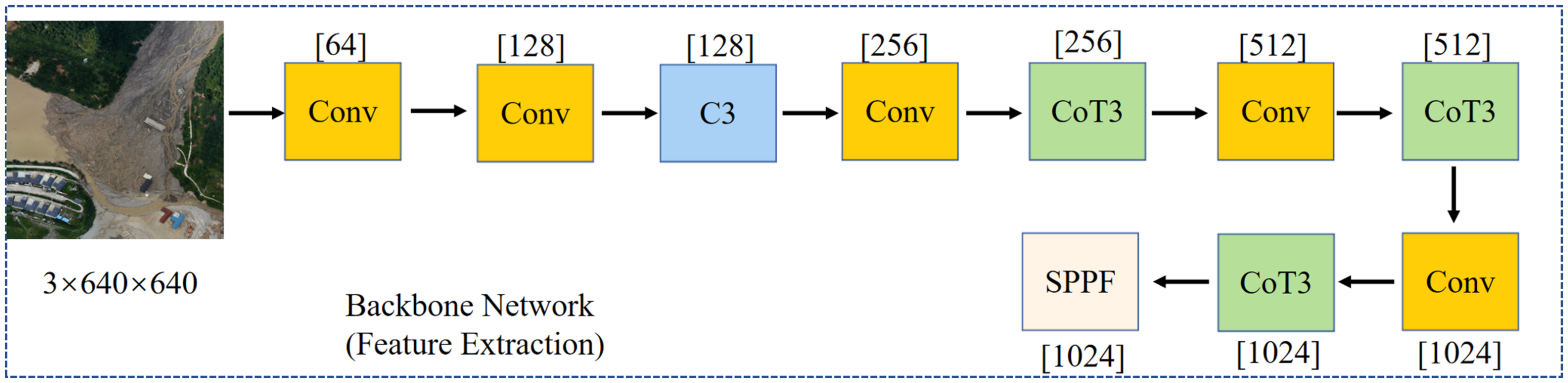
Backbone network.

This paper uses the CSPDarknet53 model of YOLOv5s as the backbone, which builds on the Darknet53 module in YOLOv3 (Redmon and Farhadi, 2018) and integrates the advantages of CSPNet (Wang et al., 2020). The CSPDark53 structure alleviates long inference times caused by gradient duplication during network optimization. At the same time, it leverages local cross-layer connections to reduce parameter count and strike a balance between speed and accuracy. Figure 2 shows the backbone network for feature extraction. The network uses alternating convolutional operations, the C3 module, and the contextual Transformer module to effectively extract and fuse feature information at each layer. The SPPF module then outputs a fixed-size feature vector. This not only enables the fusion of multi-scale features but also supports images of arbitrary size.

**Figure 3:**
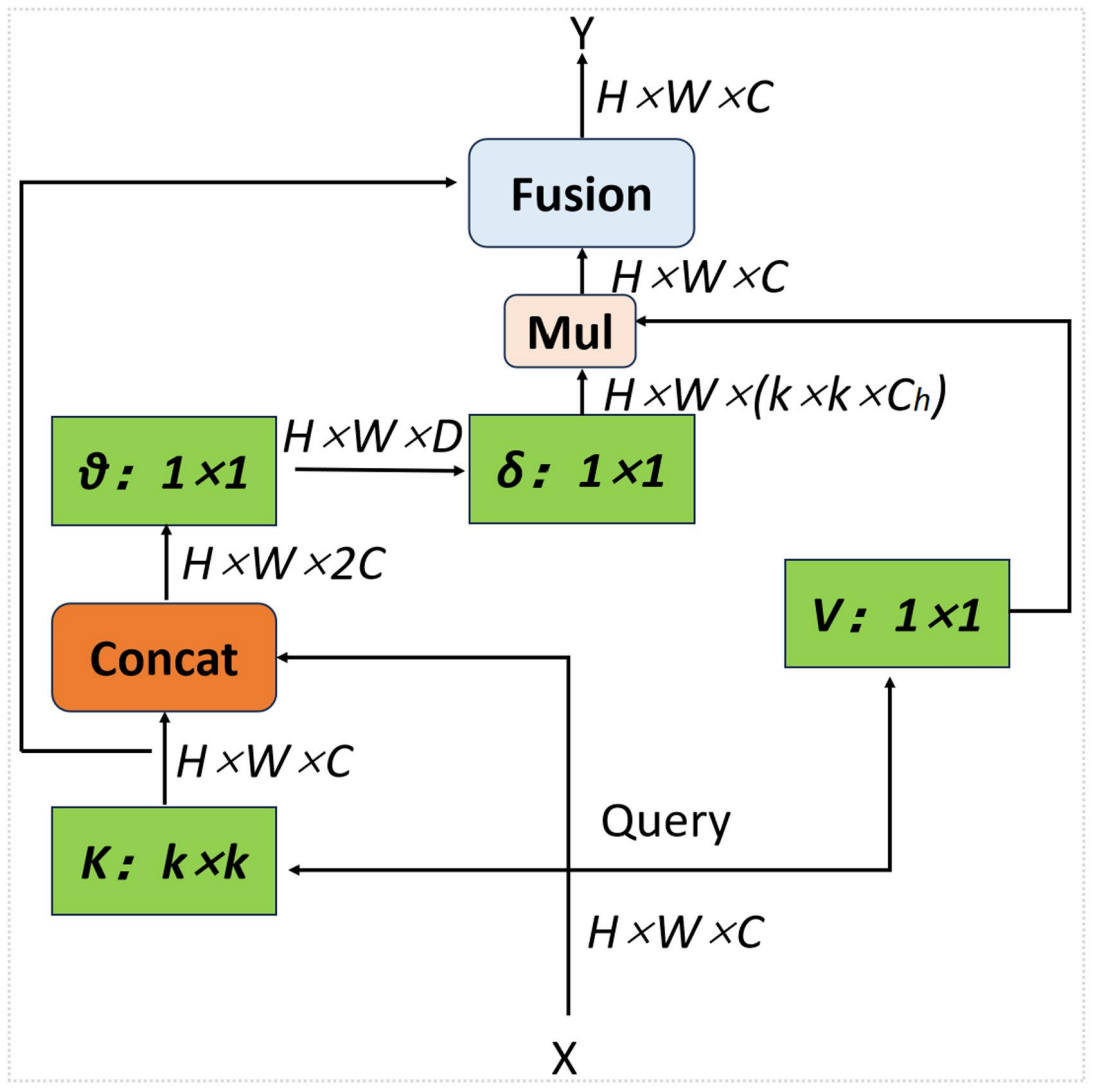
Contextual Transformer (CoT) block: the CoT module is a computational unit that enhances the representation of image features. The module can establish effective contextual relationships within the spatial and channel dimensions of the feature map, thereby improving the discriminative power and detection accuracy of target regions.

Figure 3 shows the CoT module. While traditional Transformer-based self-attention mechanisms can effectively capture feature interactions across different spatial locations, they learn all query-key relationships independently, without considering the rich contextual information between them. This severely limits the ability of self-attention mechanisms to represent visual features on two-dimensional feature maps. To address this issue, Li et al. (2022) proposed a residual network-based self-attention mechanism, namely the CoT structure shown in Figure 3. This structure effectively combines contextual information with the self-attention mechanism, aggregating features from neighboring pixels at each spatial location in the input feature map to obtain a local contextual representation that captures the target region’s structural information. Then, the local contextual information is weighted and fused with the original input features to generate an enhanced feature representation, achieving a collaborative expression of local information and global semantics. The feature map after contextual modeling retains the original information while enhancing the response of the target region, thereby improving the recognition ability of target instances of different scales and shapes. The input feature map X has spatial dimensions *H × W* and *C* channels. The convolutional module K primarily extracts local context from the input feature map, producing key features that capture neighborhood information. The concatenation module, concat, primarily fuses the original features with local contextual information, providing rich feature representations for subsequent weight calculations. The convolution module *θ* reduces the dimensionality of the concatenated features to D channels, thereby generating attention weights. The convolution module *δ* takes the projected features as input and computes the corresponding weighting coefficients for each spatial location, thereby characterizing the importance of local context within the global features. The convolution module V applies a linear transformation to the features, generating contextual information at each spatial location to produce a weighted output. The multiplication module Mul multiplies the weights δ with the features V to achieve weighted fusion of contextual information. The fusion module `Fusion` adds or concatenates the weighted contextual features to the original input X to form an enhanced feature output Y, thereby improving global context awareness while preserving the original local information.

**Figure 4:**
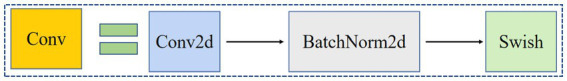
Conv module.

**Figure 5:**
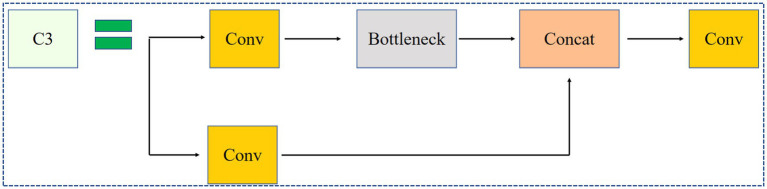
C3 module.

**Figure 6:**
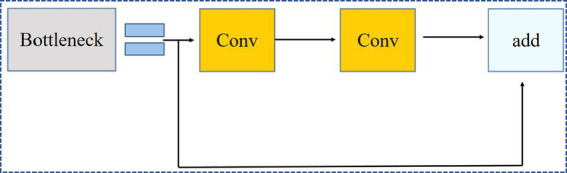
Bottleneck module.

**Figure 7:**
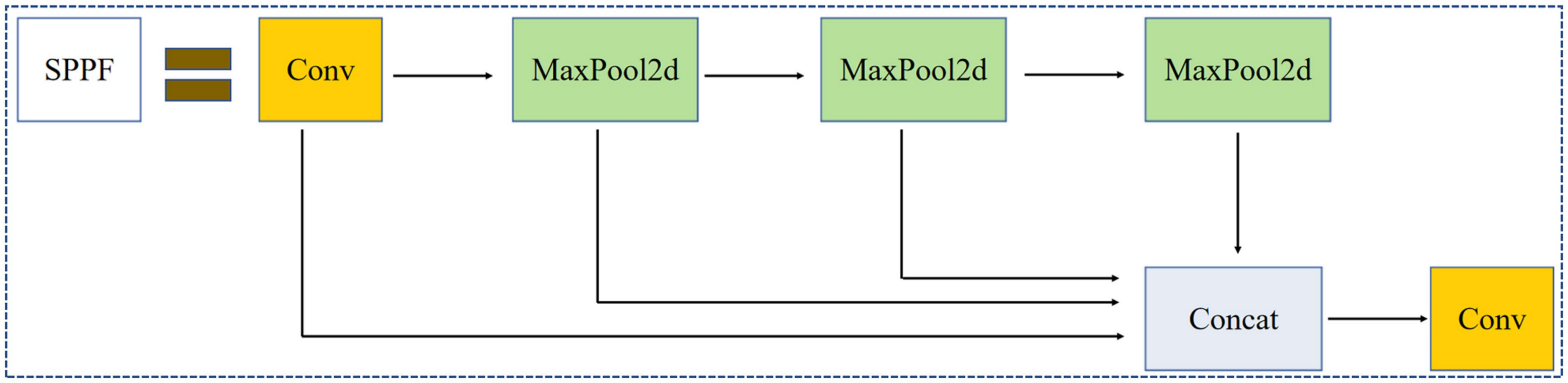
SPPF module.

Figure 4 shows the Conv module, which includes convolution operations, batch normalization, and the Swish activation function. Figure 5 shows the C3 module, which combines cross-layer feature fusion with residual networks (bottleneck modules). In the C3 module, Concat represents concatenation along the channel dimension, and the bottleneck module used is shown in Figure 6. In the bottleneck module shown in Figure 6, the addition operator represents element-wise addition. Figure 7 shows the SPPF module, where Maxpool2d denotes max pooling, and Concat denotes concatenation.

### 3.3 Feature fusion module

**Figure 8:**
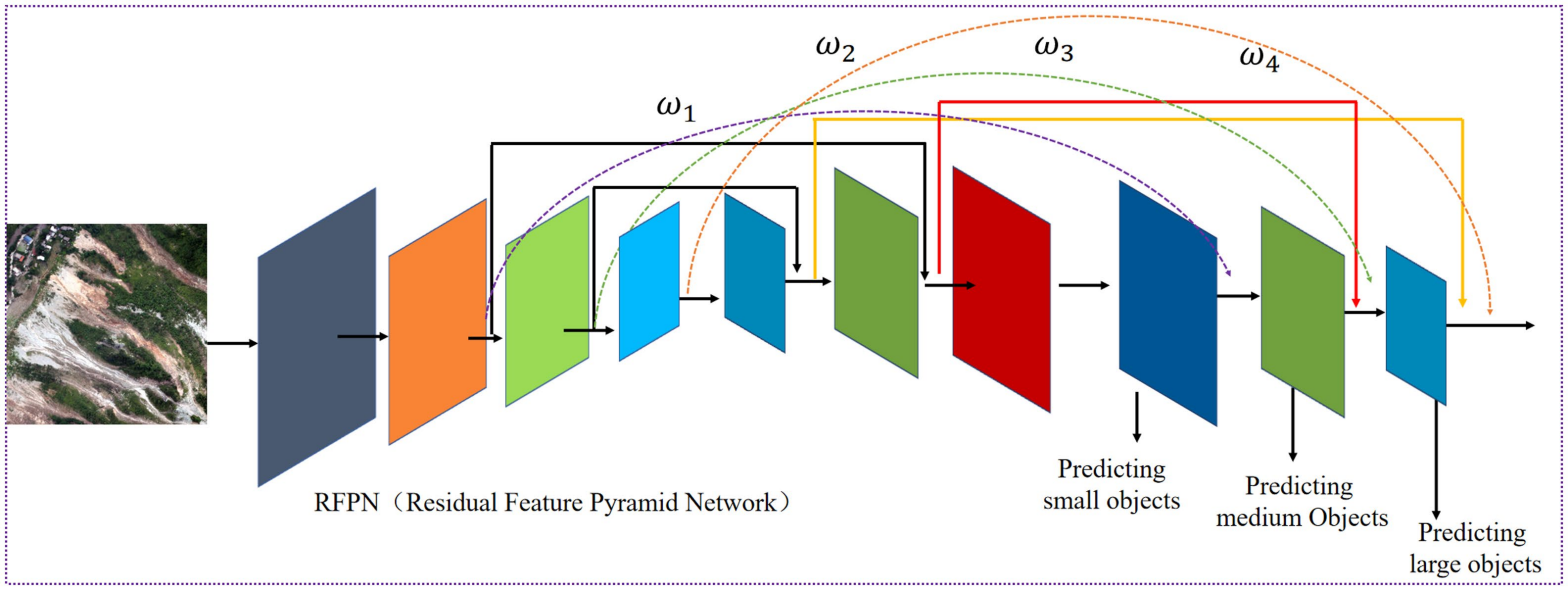
RFPN module.

Feature fusion networks can effectively integrate information across layers and scales. The idea of the weighted residual pyramid proposed in this paper for feature fusion networks is derived from the feature pyramid. Because object detection is a multi-task encompassing classification, recognition, and localization, the image’s feature information becomes more abstract and granular after being processed by a multi-layer neural network. Studies (He et al., 2015; Zhao et al., 2019) have shown that shallow features, such as edges and corners, can improve positioning accuracy, whereas abstract, deep semantic features can enhance classification performance. Since the scale of image features will continue to change after extraction by the convolutional neural network, and the convolutional neural network can be regarded as a pyramid structure, it is highly adaptable to scale transformations. Therefore, directly using the convolutional neural network’s pyramid structure can achieve multi-scale feature fusion without adding extra computational complexity. At the same time, shallow high-resolution features can enhance the accuracy of small-target detection; this is the idea behind the Feature Pyramid Network (FPN) (Lin et al., 2017). The FPN fuses multi-scale features via bottom-up and top-down horizontal connections, making full use of the pyramid structure inherent in convolutional neural networks without introducing excessive computational complexity or parameter count. Because horizontal feature information is derived from network layers at the same scale, it focuses solely on the representation of features within those layers. It cannot effectively fuse feature information from multiple layers. The feature fusion module in the basic CSPDarknet53 model introduces a bottom-up path to the FPN, thereby enhancing both high-level and low-level features to improve classification and localization capabilities, respectively. This alleviates the problem of FPN being split across layers when object sizes are relatively small (10 pixels, as specified in the PANet paper). However, PANet does not fully utilize the high-resolution backbone network’s features, and different feature layers contribute differently to the loss. To alleviate this problem, this paper proposes a weighted residual pyramid model (RFPN) that draws on PANet and ResNet, as shown in Figure 8.

As shown in Figure 8, the RFPN module combines the ideas of ResNet and the top-down and bottom-up path structures of PANet. It introduces shortcut connections at the same resolution and uses element-wise addition to fuse feature representations. This not only maximizes the enrichment of feature information in each layer but also enhances the transfer capability of feature information without significantly increasing computation or parameter count. Furthermore, because features from different network layers contribute differently during fusion, learnable weight factors are applied to the feature maps to balance their contributions at different scales. Feature fusion is then performed using a weighted method, as shown in Equation 1.


$$\text{output}=\sum_{i}\frac{w_{i}}{\varepsilon +\sum_{j}w_{j}}I_{i}$$ (1)

In the above Equation 1, *w_i_* is a learnable weight factor, and *ε* is a factor to avoid numerical instability; ε = 0.0001. It is the feature map of different levels.

### 3.4 Detection head

**Figure 9:**
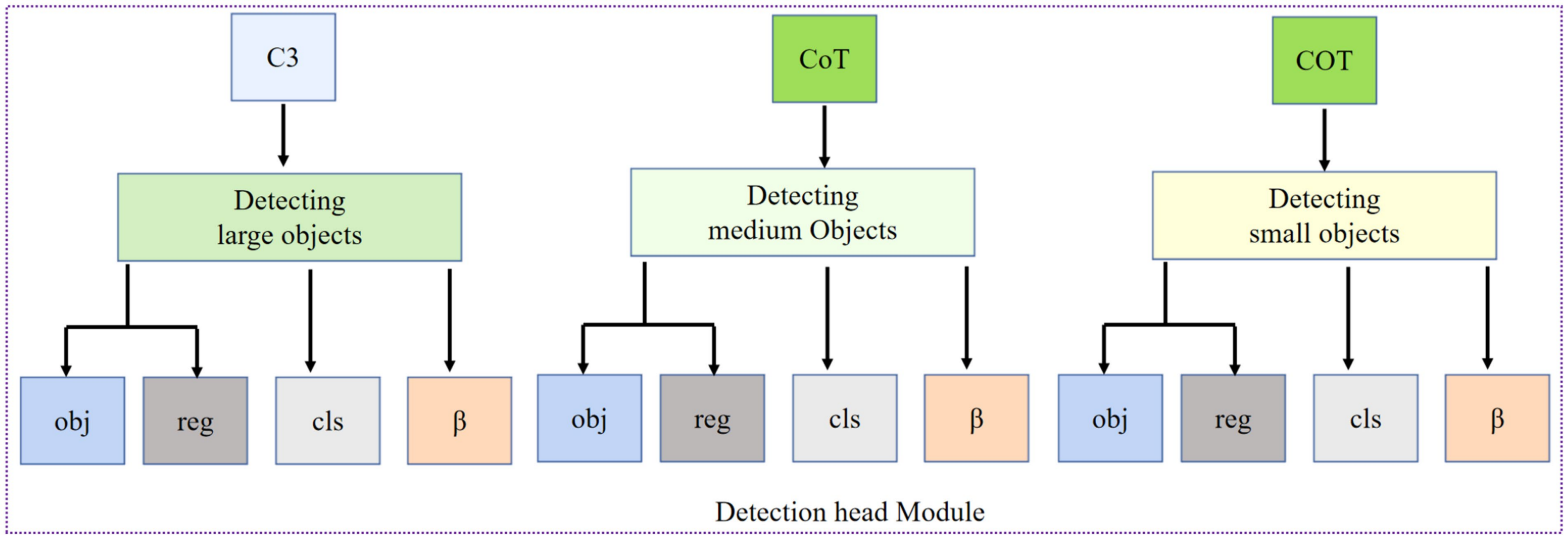
Detection head module.

The target detection task includes two subtasks: classification and positioning. In the object detection network, the feature extraction network primarily extracts key features layer by layer, thereby improving the model’s robustness and accuracy. The detection network must also produce classification and localization outputs. The detection network’s primary function is to perform predictions based on the output features of the feature fusion network. To address the multi-angle and multi-source background characteristics of remote sensing imagery, this paper employs a rotation-frame detection method to detect ground-object instances. Drawing on the data annotation methods of the YOLO family of methods, a rotation angle *β* is added to the five-parameter long edge definition method, enabling the detection module to output directional offset information for ground object instances simultaneously. The detection module is shown in Figure 9.

**Figure 10:**
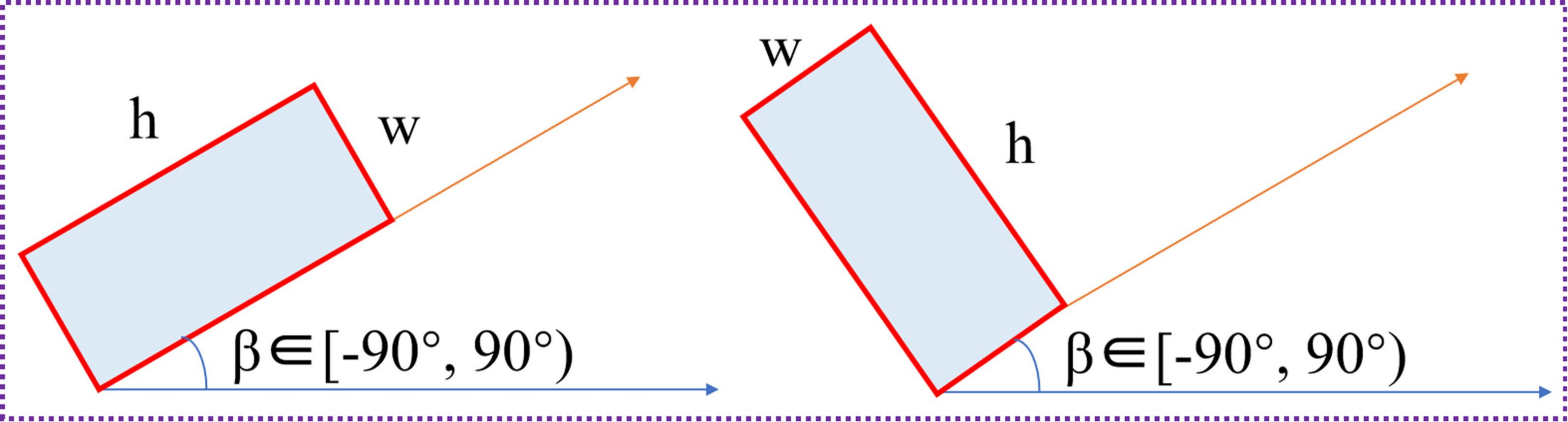
Long-side based definition.

In Figure 9, three output branches are used to detect large, medium, and small targets, respectively. Since the feature scale of the remote sensing image will continue to change after passing through the feature extraction network and the feature fusion network, the receptive field of each pixel in the small feature map is extensive, and the semantic information is abstract, which is conducive to the detection of large target instances; the receptive field of each pixel in the large feature map is small, and it contains rich information such as corners and edges, which is conducive to the detection of small target instances; the medium-scale feature map is used to detect medium-sized target instances. The detection network outputs the classification probability (*cls* in Figure 9), confidence (*obj* in Figure 9), regression coordinates (*reg* in Figure 9) (*x, y, w, h*), and rotation angle *β* (the value range is −90° ~ 90°); the regression coordinates and the rotation angle constitute the rotation prediction box (*x, y, w, h, β*) of the long edge definition method. The rotation angle *β* is obtained by the 5-parameter long edge definition method (Yang and Yan, 2020), as shown in Figure 10.

As shown in Figure 10, the five-parameter method uses five variables to achieve the positioning of the rotating bounding box, namely (*x, y, w, h, β*), where (*x, y*) is the center point coordinate, (*w, h*) is the width and height, and *β* is the angle parameter; the long side definition method stipulates that the clockwise direction is a positive angle and the counterclockwise direction is a negative angle. The range of the defined angle parameter is *β* ∈ [*−90°, 90°*). This method describes the short side as the width and the long side as the height.

### 3.5 Improved loss function

The loss function, a crucial component in the training process of object detection models, measures the discrepancy between the model’s predictions and the ground-truth annotations, thereby guiding the optimization of model parameters. During training, the model calculates the prediction error via forward propagation and updates its parameters via backpropagation to minimize the overall loss. Therefore, the design of the loss function directly affects the model’s performance. Given the arbitrary distribution of target instances in remote sensing imagery, traditional horizontal bounding boxes struggle to describe their geometry accurately and often introduce redundant background information, thereby degrading model performance. Therefore, this paper designs a multi-task rotated bounding box loss function to mitigate the imbalance in the contribution of different target instances to the regression task. The loss function consists of four parts: classification loss, confidence loss, localization loss, and angle loss, used to constrain the category, target confidence, location regression, and orientation, respectively. In multi-task loss, different constraints contribute differently to the overall optimization. Using uniform weights during optimization can easily lead to bias toward one task during training, affecting overall model performance. Therefore, this paper introduces weighting factors into the loss function to balance the contributions of each loss term and mitigate the imbalance in the multi-task joint optimization process. The loss function formula is defined in Equation 2.


$$\text{Loss}=\lambda_{\mathrm{reg}}L_{\mathrm{reg}}+\lambda_{\mathrm{cls}}L_{\mathrm{cls}}+\lambda_{\beta }L_{\beta }+\lambda_{\mathrm{obj}}L_{\mathrm{obj}}$$ (2)

In the above Equation 2, *L_reg_* represents the regression loss, *L_cls_* represents the classification loss, *L_β_* represents the rotation angle loss, *L_obj_* represents the confidence loss, and *λ* represents the weight of each subtask loss. Different values are assigned according to the task’s contribution to the loss. In the experiment, *λ_reg_* = 0.05; *λ_cls_* = 0.5; *λ_obj_* = 1; *λ_β_* = 0.5. For classification loss, confidence loss, and rotation angle loss, the binary cross-entropy loss function is used, as shown in Equations 3–7.


$$L_{\mathrm{cls}}=-w_{\mathrm{cls}}[\begin{array}{l} p_{\mathrm{cls}}*y_{\mathrm{cls}}*\log(\sigma (x_{\mathrm{cls}}))\\ +(1-y_{\mathrm{cls}})*\log(1-\sigma (x_{\mathrm{cls}})) \end{array}]$$ (3)

$$L_{\beta }=-w_{\beta }[p_{\beta }*y_{\beta }*\log(\sigma (x_{\beta }))+(1-y_{\beta })*\log(1-\sigma (x_{\beta }))]$$ (4)

$$L_{\mathrm{obj}}=-w_{\mathrm{obj}}[\begin{array}{l} p_{\mathrm{obj}}*y_{\mathrm{obj}}*\log(\sigma (x_{\mathrm{obj}}))\\ +(1-y_{\mathrm{obj}})*\log(1-\sigma (x_{\mathrm{obj}})) \end{array}]$$ (5)

$$\log(\sigma (x))=\log(\frac{1}{1+e^{-x}})$$ (6)

$$\log(1-\sigma (x))=\log(\frac{e^{-x}}{1+e^{-x}})\;$$ (7)

Equations 3–7 provide the calculation methods for the confidence loss, rotation angle loss, and classification loss. In the formulas, *w_cls_*, *w_β_*, *w_obj,_* and *p_cls_*, *p_β_*, *p_obj_* are weight factors, which are taken as 1 in the experiment; *y_cls_*, *y_obj_*, *y_β_* are the label values of the input samples; *x_cls_*, *x_β_*, *x_obj_* are the class probability values, angle values, and confidence values predicted by the model. Equations 8–13 define the regression loss.


$$L_{\mathrm{reg}}=1-\text{CIoU}$$ (8)

$$\text{CIoU}=\mathrm{IoU}-(\frac{d^{2}}{c^{2}}+\alpha \nu )$$ (9)

$$\mathrm{IOU}=\frac{S_{\text{intersection}}}{S_{\text{union}}}$$ (10)

$$\nu =\frac{4}{\pi^{2}}{\left(\arctan\frac{w^{\mathrm{gt}}}{h^{\mathrm{gt}}}-\arctan\frac{w}{h}\right)}^{2}$$ (11)

$$\alpha =\frac{\nu }{(1-\mathrm{IoU})+\nu }$$ (12)

$$d=\sqrt{{\left(x_{2}-x_{1}\right)}^{2}+{\left(y_{2}-y_{1}\right)}^{2}}$$ (13)

**Figure 11:**
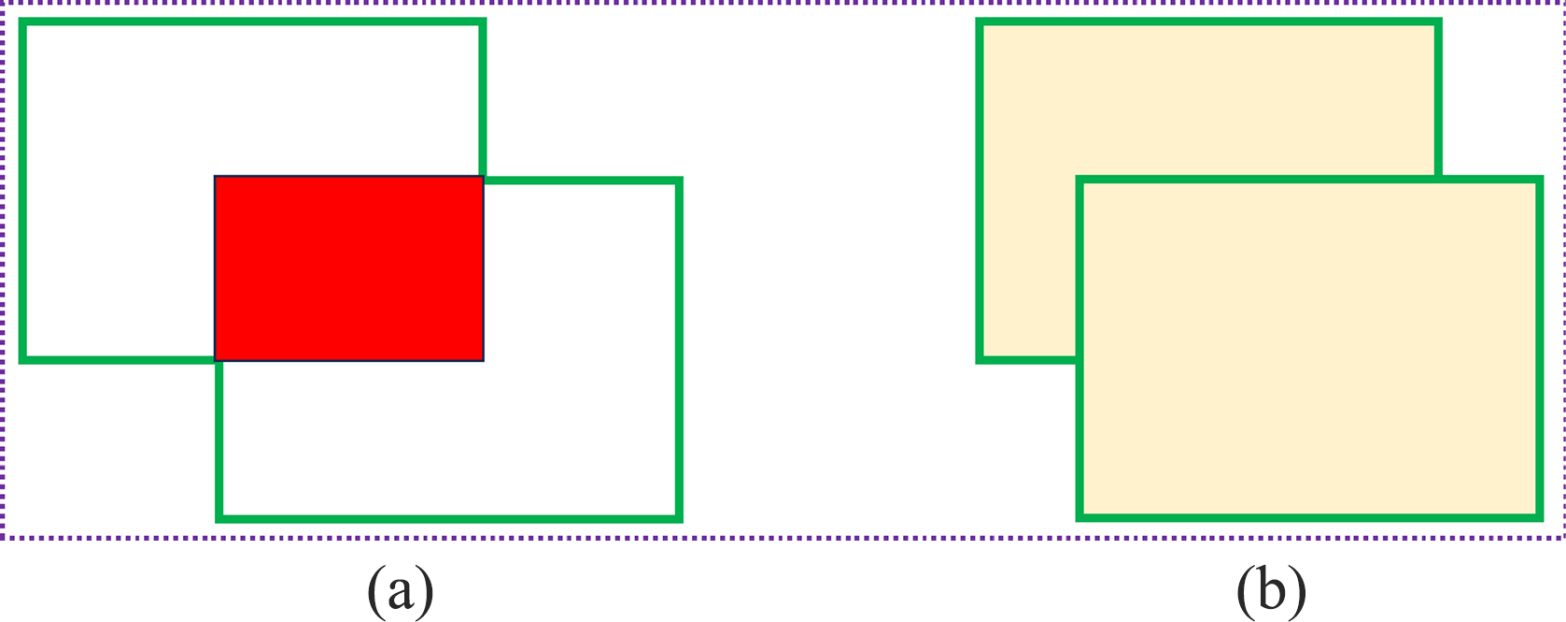
Intersection and union: **(a)** Intersection; **(b)** Union.

**Figure 12:**
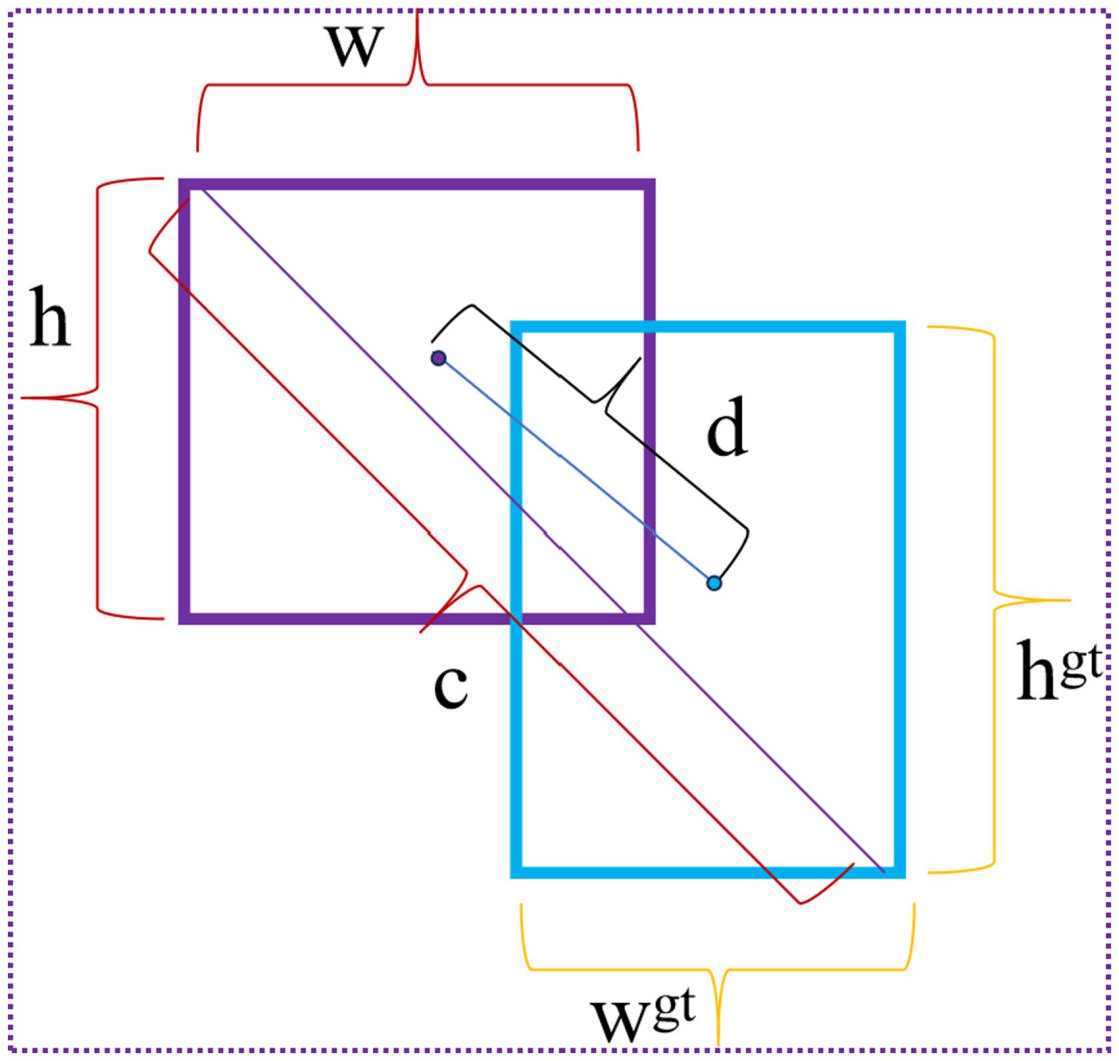
Distance between annotation box and prediction box centers and minimum enclosing rectangle diagram.

Equations 8–13 give the calculation formulas for the improved regression loss function. In Equation 10, IoU (Intersection over Union) is the intersection-over-union ratio; that is, the ratio of the intersection and union of the model’s predicted bounding box and the actual bounding box, where the numerator *S_intersection_* represents the area of the intersection, and the denominator *S_union_* represents the area of the union. The intersection and union are shown in Figure 11. In Equation 11, *w* and *h* represent the width and height of the predicted bounding box, respectively, and *w^gt^* and *h^gt^* represent the width and height of the labeled bounding box, respectively; *c* represents the diagonal length of the minimum circumscribed rectangle of the predicted bounding box and the true labeled bounding box. The (*x_i_, y_i_*) in Equation 13 are the coordinates of the center points of the predicted bounding box and the actual bounding box; d represents the Euclidean distance between the center points of the predicted bounding box and the actual labeled bounding box, and *α* is the intermediate conversion factor. The schematic diagram is shown in Figure 12.

### 3.6 Datasets

Given the lack of large-scale, well-annotated datasets for remote sensing image target detection, Xia et al. (2018) released the DOTA (Dataset for Object Detection in Aerial Images). This dataset contains numerous target instances with randomly distributed orientations, including complex scene information. There are two annotation methods: horizontal box annotation and rotation box annotation.

#### 3.6.1 DOTAv1.0 dataset

**Figure 13:**
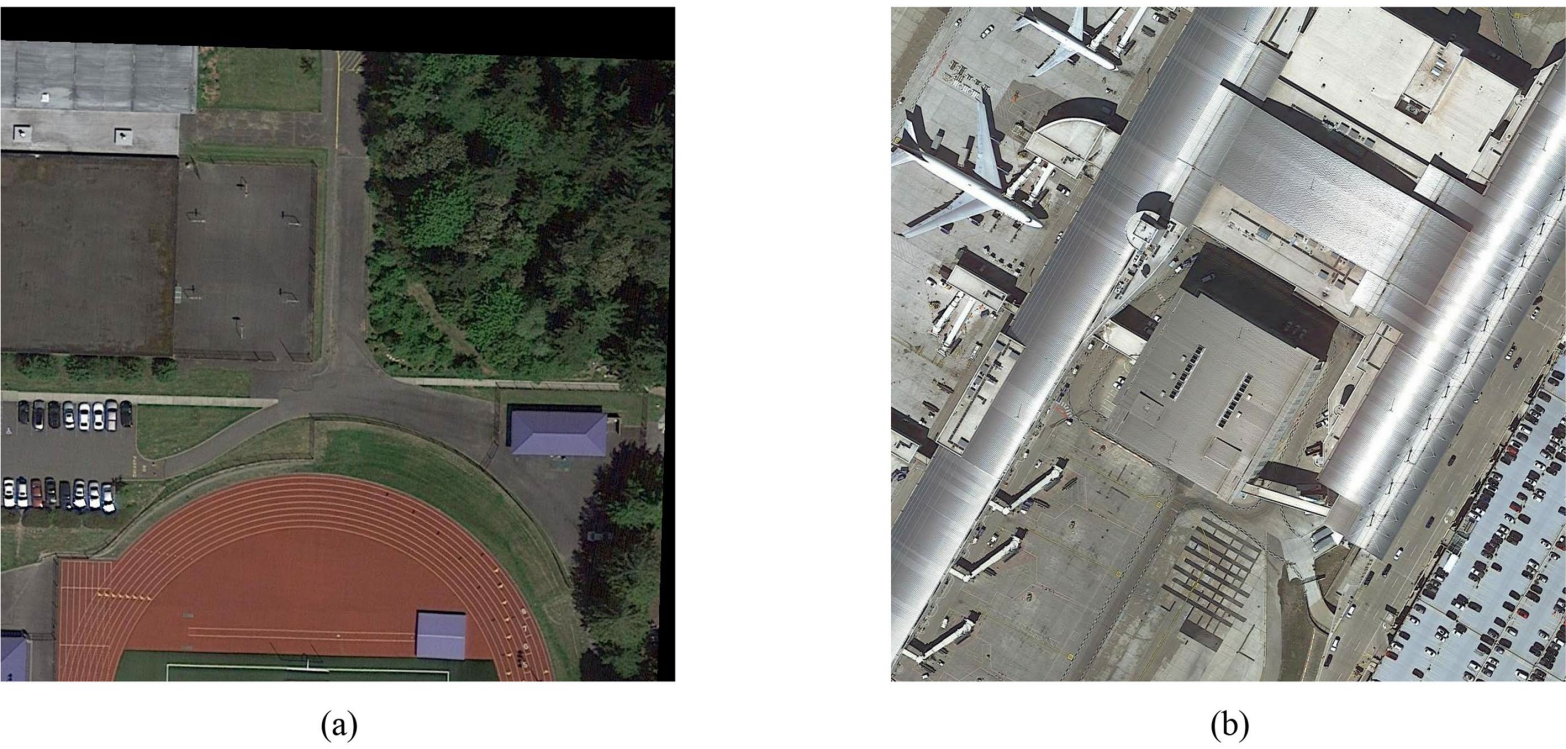
DOTAv1.0 dataset examples: **(a)** scenario 1; **(b)** scenario 2.

The DOTAv1.0 dataset comprises 2,806 images at a resolution of 4,000 × 4,000, featuring target instances of varying sizes, orientations, and shapes. It includes 15 categories and 188,282 instances. The category instances are truck, football field, ship, roundabout, tennis court, oil tank, track and field, bridge, car, airplane, baseball field, port, helicopter, swimming pool, and basketball court. The dataset sample is shown in Figure 13.

#### 3.6.2 DOTAv1.5 dataset

**Figure 14:**
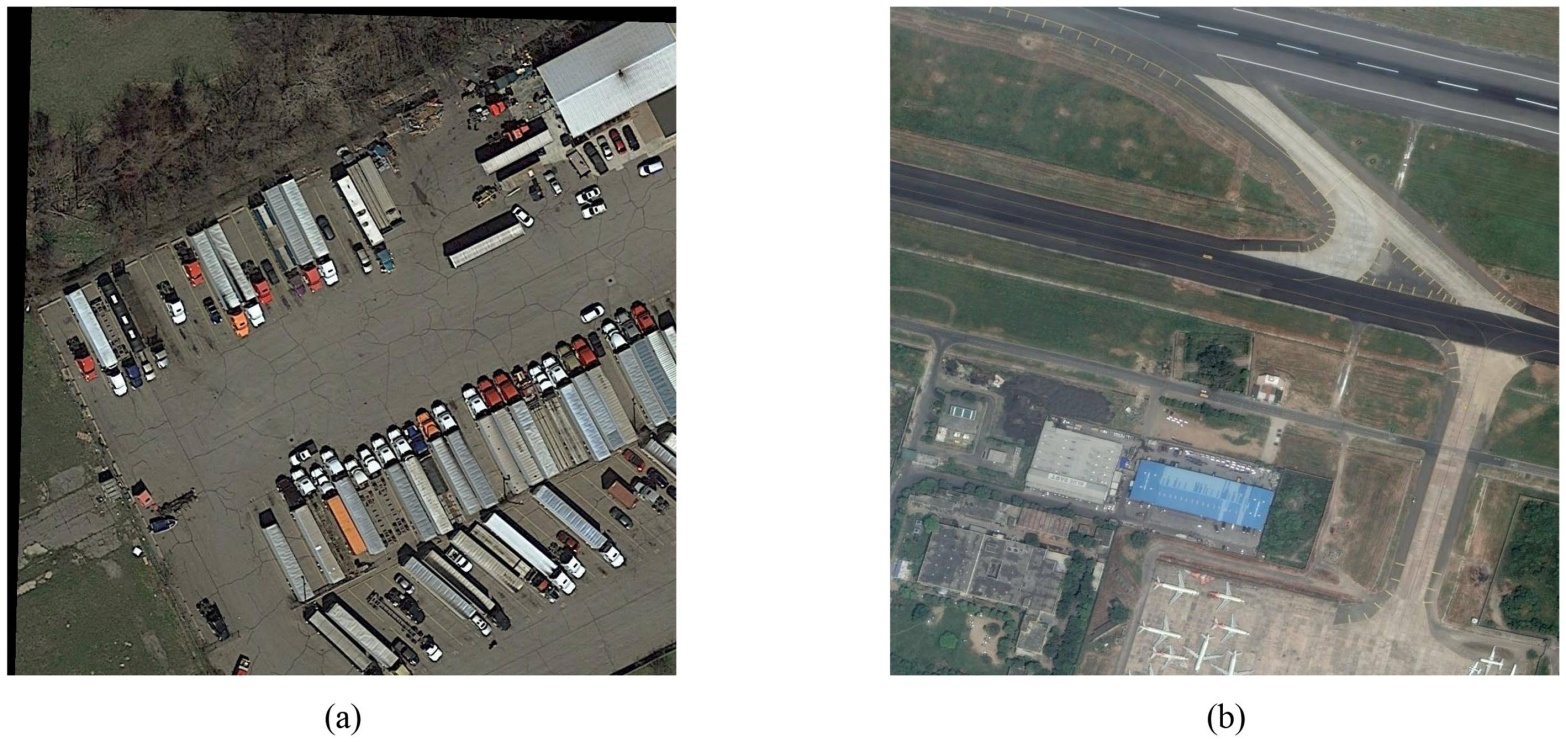
DOTAv1.5 dataset examples: **(a)** scenario 1; **(b)** scenario 2.

The DOTAv1.5 dataset introduces a container crane category, building upon the previous version and incorporating new instance annotations. There are 16 categories in total, including trucks, football fields, ships, roundabouts, tennis courts, oil tanks, athletic fields, bridges, cars, airplanes, baseball fields, ports, helicopters, swimming pools, basketball courts, and container cranes, for a total of 403,318 instances. The dataset instance diagram is shown in Figure 14.

## 4 Experiments and results analysis

The experimental part begins with performance comparisons and ablation studies, followed by some failed exploratory experiments, and concludes with real disaster-scene detection. The comparisons and ablation experiments use two datasets, DOTA v1.0 and DOTA v1.5, introduced in Section 3.6. The experiments were conducted in the PyTorch deep learning framework on Ubuntu 20.04, using a CUDA 11.2 accelerator. The hardware environment consisted of an Intel E5-2680 v4 CPU and a 32GB Tesla V100 NVIDIA graphics card. The input remote sensing image for the detection network was 640 × 640 pixels. During model training, the initial learning rate was set to 0.01, and a cosine annealing schedule was used for dynamic adjustment, with a lower bound of 0.002. The weight decay coefficient was set to 0.0005, the momentum to 0.937, the number of training iterations to 300, and the batch size to 64. All experiments were conducted under the same training and evaluation settings to ensure the fairness and comparability of the results.

### 4.1 Evaluation methodology

This paper primarily discusses a bounding-box-based remote sensing image and an object-detection model. The following primarily introduces the evaluation method used in the experiment.

(1) Precision P: Also known as the precision rate, it represents the correct proportion of the predicted positive class, that is, the proportion of correctly predicted positive samples to the predicted positive samples, as shown in Equations 14, 15.

$$P=\frac{\mathrm{TP}}{P^{\prime }}$$ (14)

$$P^{\prime }=\mathrm{TP}+\mathrm{FP}$$ (15)

(2) Recall rate R (Recall): also known as the actual positive sample rate, represents the ratio of correctly predicted positive samples to actual positive samples, as shown in Equations 16, 17.

$$R=\frac{\mathrm{TP}}{\text{Positive}}$$ (16)

Positive=TP+FN (17)

In Equations 14–17, *TP* represents the accuracy rate of the predicted positive samples; FP represents the error rate of the predicted positive samples; *Positive* represents the actual positive samples; and *P′* represents the predicted positive samples.

AP (Average Precision): The average precision for a category. This curve is plotted using different confidence thresholds to obtain different recall and precision values. The curve has recall on the horizontal axis and precision on the vertical axis. The area under the curve is the AP value, also known as the area under the Precision-Recall curve. A higher AP value indicates better model performance. The calculation method is shown in Equation 18. Equation 19 can also be used for approximate calculations.


$$AP_{\text{type}}=\int_{0}^{1}p(r)\mathrm{dr}$$ (18)

$$AP_{\mathrm{tpy}e_{i}}=\frac{1}{N}\sum_{j=1}^{N}\text{Precisio}n_{j}$$ (19)

In Equation 19, N represents the total number of samples in the i-th class, and j represents the j-th sample in the i-th class.

(3) mAP (mean average precision): The average precision of all categories, that is, the sum of all category APs divided by the number of categories, as shown in Equation 20.

$$\;\;\;\mathrm{mAP}=\frac{\sum_{i=1}^{N}AP_{\mathrm{typ}e_{i}}}{N}$$ (20)

In Equation 20, i represents the i-th category, and N represents the total number of the category.

### 4.2 Comparative experiments and discussion of results

This section presents the experimental results of the proposed model on the DOTAv1.0 and DOTAv1.5 datasets (Tables 1, 2) and compares them with mainstream models. The results demonstrate the superiority and effectiveness of our model. Table 1 presents the experimental results of the model proposed in this paper, along with those of one-stage and two-stage models, on the DOTAv1.0 dataset. Table 2 presents the experimental results of the model proposed in this paper, along with those of one-stage and two-stage models, on the DOTAv1.5 dataset.

**Table 1:** Comparative results on the DOTAv1.0 dataset.

Network model	Network layers	Parameters×10^6^	GFLOPs	FPS	P(all)	R(all)	mAP@0.5
Faster R-CNN	40	137.098724	370.21	16.7	0.496	0.813	0.73
YOLOv5s	213	7.05058	15.9	276	0.715	0.673	0.676
YOLOv5m	290	20.909508	48	159	0.787	0.678	0.715
YOLOv8n	168	3.008573	8.1	300	0.81	0.693	0.744
YOLOx	360	8.94	13.39	25	0.829	0.647	0.7495
RT-DERT-l	502	32.014565	103.5	31	0.755	0.688	0.731
CoT_WRPNet (Ours)	303	7.49732	17.3	50	0.755	0.718	0.754
YOLOv9m	151	20.023837	76.6	43	0.791	0.763	0.787
YOLOv10m	136	15.321853	58.9	31	0.829	0.734	0.792

**Table 2:** Comparative results on the DOTAv1.5 dataset.

Network model	Network layers	Parameters×10^6^	GFLOPs	FPS	P(all)	R(all)	mAP@0.5
Faster R-CNN	40	137.099	370.21	11	0.787	0.614	0.695
YOLOv5s	213	7.053277	15.9	269	0.804	0.598	0.64
YOLOv5m	290	20.913549	48.1	138	0.769	0.669	0.704
YOLOv8n	168	3.151904	8.7	328	0.767	0.65	0.693
YOLOx	360	8.94	13.39	21	0.872	0.591	0.691
RT-DERT-l	506	32.01662	103.5	27	0.749	0.64	0.688
CoT_WRPNet (Ours)	303	7.500089	17.3	48	0.689	0.69	0.714
YOLOv9m	151	20.02456	76.6	40	0.83	0.697	0.748
YOLOv10m	136	15.322432	58.9	35	0.843	0.688	0.757

As shown in Table 1, the Faster R-CNN model has approximately 18.3 times as many parameters, 21.4 times the computational cost (GFLOPs), and 0.334 times the frame rate (PFS) as the proposed CoT_WRPNet model. Based on the speed and space-complexity comparison, the Faster R-CNN model is significantly slower than the proposed model and has a substantially larger parameter count. However, the average class accuracy (mAP@0.5) at an IoU threshold of 0.5 in the last column shows that the proposed CoT_WRPNet model is 2.4% higher. This phenomenon indicates that the CoT_WRPNet model can achieve higher accuracy while maintaining detection speed and space complexity. This is because Faster R-CNN employs a two-stage detection architecture: first, generating candidate regions, then performing classification and regression. While this ensures detection accuracy, it significantly increases the number of parameters and computational cost and decreases inference speed. In contrast, CoT_WRPNet employs lightweight convolutions and context transformers to optimize feature extraction and adopts an end-to-end single-stage detection strategy. This allows the model to significantly reduce computational complexity while maintaining high accuracy, thereby improving FPS. Compared to the YOLOv5s model, our proposed model has slightly more network layers, more parameters, and slightly higher GFLOPs, but slightly lower FPS (although still meeting real-time requirements). This is because, while YOLOv5s’ lightweight design improves speed, it sacrifices some feature representation, resulting in a 7.8% lower mAP@0.5 and P and R values decreasing by 4 and 4.5%, respectively. Compared to YOLO5m, the model in this paper has about 2.78 times higher parameter count and GFLOPs. However, its mAP@0.5 is still 3.9% lower, indicating that excessively increasing the network size does not directly improve accuracy. Compared to the CoT_WRPNet model, YOLOv8n has fewer network layers, parameters, and GFLOPs, resulting in faster inference and higher FPS. However, its mAP@0.5 is 1% lower than the CoT_WRPNet model. This indicates that although YOLOv8n employs a lightweight design to improve speed, its feature extraction and fusion capabilities are relatively weak, leading to reduced detection accuracy. In contrast, CoT_WRPNet, through multi-scale feature fusion and an optimized attention mechanism, improves object recognition accuracy while maintaining real-time inference. Therefore, it maintains a higher mAP@0.5 while balancing speed, parameter count, and space complexity, fully demonstrating the model’s comprehensive advantages in accuracy and efficiency. Compared with the CoT_WRPNet model, YOLOx has 57 more network layers and 1.19 times as many parameters. Its GFLOPs are approximately 0.77 times those of our model, while its FPS are half of our model. However, its mAP@0.5 is 0.45% lower than that of the CoT_WRPNet model. This indicates that although YOLOx uses multi-layer optimization, it does not completely surpass CoT_WRPNet’s capabilities in feature extraction and fusion. Therefore, our model is slightly more accurate. Compared to the RT-DETR-1 model, CoT_WRPNet improves mAP@0.5 by 2.3%. In terms of model complexity, CoT_WRPNet reduces the number of network layers by 199, has approximately 0.23 times the number of parameters of RT-DETR-1, about 0.17 times the GFLOPs, and about 1.6 times the FPS. This demonstrates that, while significantly reducing model complexity, CoT_WRPNet still achieves higher detection accuracy, indicating greater efficiency and effectiveness in feature extraction and multi-scale fusion. Compared to the YOLOv9m model, CoT_WRPNet’s mAP@0.5 is 75.4%, lower than YOLOv9m’s 78.7%. In terms of model complexity, CoT_WRPNet has 152 more network layers than YOLOv9m. Still, its parameter count is only 0.37 times that of YOLOv9m, its GFLOPs are approximately 0.23 times, and its FPS is approximately 1.16 times higher. This shows that, although there is a difference in detection accuracy, CoT_WRPNet significantly reduces parameter size and computational cost while maintaining high inference speed, making it a practically advantageous choice for resource-constrained applications or those with stringent real-time requirements. Compared to YOLOv10m, CoT_WRPNet’s mAP@0.5 is 75.4%, lower than YOLOv10m’s 79.2%. In terms of model complexity, CoT_WRPNet increases the number of network layers by 167. Still, its parameter count is only 0.49 times that of YOLOv10m, its GFLOPs are approximately 0.29 times, and its FPS is approximately 1.61 times higher. This shows that, although there is a difference in detection accuracy, CoT_WRPNet has significantly lower computational cost and parameter count, and an advantage in inference speed, indicating that this model performs better in terms of computational efficiency and real-time performance. This, to some extent, suggests that the proposed method achieves a good balance between performance and computational cost. Therefore, experimental results on the DOTAv1.0 dataset demonstrate that CoT_WRPNet effectively reduces model parameters and computational cost while maintaining high detection accuracy and fast inference speed, thereby exhibiting good overall performance.

As shown in Table 2, the Faster R-CNN model has approximately 18.3 times the number of parameters, 21.4 times the computational cost (GFLOPs), and 0.229times the frame rate (PFS) of the proposed CoT_WRPNet model. Based on the speed and space-complexity comparison, the Faster R-CNN model is significantly slower than the proposed model and has a substantially larger parameter count. However, the average class accuracy (mAP@0.5) at an IoU threshold of 0.5 in the last column shows that the proposed CoT_WRPNet model is 1.9% higher. This phenomenon indicates that the CoT_WRPNet model can achieve higher accuracy while maintaining detection speed and space complexity. This is because Faster R-CNN employs a two-stage detection architecture: first, generating candidate regions, then performing classification and regression. While this ensures detection accuracy, it significantly increases the number of parameters and computational cost and decreases inference speed. In contrast, CoT_WRPNet employs lightweight convolutions and context transformers to optimize feature extraction and adopts an end-to-end single-stage detection strategy. This allows the model to significantly reduce computational complexity while maintaining high accuracy, thereby improving FPS. Compared to the YOLOv5s model, our proposed model has slightly more network layers, more parameters, and slightly higher GFLOPs, but slightly lower FPS (although still meeting real-time requirements). This is because while YOLOv5s’ lightweight design improves speed, it sacrifices some feature representation, resulting in a 7.4% lower mAP@0.5 and a 10.1% decrease in R. Compared to YOLO5m, its parameter count and GFLOPs are about 2.78 times higher than those of the model in this paper. However, its mAP@0.5 is still 1% lower, indicating that excessively increasing the network size does not directly improve accuracy. Compared to the CoT_WRPNet model, YOLOv8n has fewer network layers, parameters, and GFLOPs, resulting in faster inference and higher FPS. However, its mAP@0.5 is 2.1% lower than the CoT_WRPNet model. This indicates that although YOLOv8n employs a lightweight design to improve speed, its feature extraction and fusion capabilities are relatively weak, leading to reduced detection accuracy. In contrast, CoT_WRPNet, through multi-scale feature fusion and an optimized attention mechanism, improves object recognition accuracy while maintaining real-time inference. Therefore, it maintains a higher mAP@0.5 while balancing speed, parameter count, and space complexity, fully demonstrating the model’s comprehensive advantages in accuracy and efficiency. Compared with the CoT_WRPNet model, YOLOx has 57 more network layers and 1.19 times as many parameters. Its GFLOPs are approximately 0.77 times ours, while its FPS are 0.435 times ours. However, its mAP@0.5 is 2.3% lower than that of the CoT_WRPNet model. This indicates that although YOLOx uses multi-layer optimization, it does not completely surpass CoT_WRPNet’s capabilities in feature extraction and fusion. Therefore, our model is slightly more accurate. Compared to the RT-DETR-1 model, CoT_WRPNet improves mAP@0.5 by 2.6%. In terms of model complexity, CoT_WRPNet reduces the number of network layers by 203, has approximately 0.234 times the number of parameters of RT-DETR-1, about 0.17 times the GFLOPs, and about 1.8 times the FPS. This demonstrates that CoT_WRPNet significantly reduces model parameters and computational overhead while improving detection accuracy and inference speed, thereby exhibiting high computational efficiency. Compared to the YOLOv9m model, CoT_WRPNet’s mAP@0.5 is 71.4%, lower than YOLOv9m’s 74.8%. In terms of model complexity, CoT_WRPNet has 152 more network layers, but its parameter count is only 0.375 times that of YOLOv9m, its GFLOPs are approximately 0.23 times, and its FPS is approximately 1.2 times higher. This shows that although the detection accuracy is slightly lower, CoT_WRPNet has a significantly smaller parameter count and computational cost, and its inference speed is faster, with lower overall computational overhead. Compared to YOLOv10m, CoT_WRPNet’s mAP@0.5 is 71.4%, lower than YOLOv10m’s 75.7%. In terms of model complexity, CoT_WRPNet has 167 more network layers. Still, its parameter count is only 0.49 times that of YOLOv10m, its GFLOPs are approximately 0.29 times, and its FPS is approximately 1.37 times higher. This demonstrates that, although detection accuracy is slightly lower, CoT_WRPNet significantly reduces the number of parameters and computational cost, while achieving faster inference and lower overall computational overhead. Therefore, experimental results on the DOTAv1.5 dataset show that CoT_WRPNet effectively reduces model parameters and computational cost while maintaining high detection accuracy and relatively fast inference, demonstrating good overall performance.

### 4.3 Ablation experiments and results discussion

#### 4.3.1 Experimental results and analysis of the DOTAv1.0 dataset

This experiment was conducted on the DOTAv1.0 dataset introduced in Section 3.6. The DOTAv1.0 dataset comprises 15 categories, with irregular distributions of target object instances and complex backgrounds. In this experiment, we used P, R, and mAP@0.5 to evaluate the model’s detection accuracy, and measured its complexity and speed using parameter count, FLOPs, and FPS. The corresponding ablation test results are listed in Table 3. In Table 3, YOLOv5s represents the original baseline model; RFPN_YOLOv5s is a model obtained by introducing a residual feature pyramid structure on top of YOLOv5s; R_YOLOv5s is a model using rotated bounding boxes to label targets; and WRPNet is a model constructed by further combining a weighted residual pyramid structure on top of rotated bounding box labeling. Based on this, a contextual Transformer module is introduced into different models to enhance their ability to capture contextual information. CoT_YOLOv5s, CoT_RFPN_YOLOv5s, and CoT_R_YOLOv5s denote models obtained by integrating this module into YOLOv5s, RFPN_YOLOv5s, and R_YOLOv5s, respectively. CoT_WRPNet is the model obtained by further incorporating a contextual Transformer module into WRPNet and is also the final model proposed in this paper.

**Table 3:** Ablation results on the DOTAv1.0 dataset.

Method model	YOLOv5s	RFPN_YOLOv5s	R_YOLOv5s	WRPNet	CoT_YOLOv5s	CoT_RFPN_YOLOv5s	CoT_R_YOLOv5s	CoT_WRPNet (Ours)
Baseline (CSPDarkNet53)	√	√	√	√	√	√	√	√
RFPN		√		√		√		√
Rotating Frame			√	√			√	√
CoT					√	√	√	√
Network Layers	213	213	213	213	287	287	303	303
Parameters×10^6^	7.05058	7.050588	7.53604	7.536048	6.341972	6.341980	7.497384	7.497392
GFLOPs	15.9	15.9	17.4	17.4	15.5	15.5	17.3	17.3
FPS	276	284	208	207	49	50	58	50
P(all)	0.715	0.74	0.764	0.76	0.762	0.808	0.75	0.755
R(all)	0.673	0.667	0.664	0.677	0.675	0.695	0.674	0.718
mAP@0.5	0.676	0.681	0.706	0.711	0.713	0.75	0.716	**0.754**

The bold values indicate the best mAP@0.5.

Table 3 presents a performance comparison of the various models. Structurally, YOLOv5s, RFPN_YOLOv5s, R_YOLOv5s, and WRPNet maintain the same number of network layers, while the number of network layers increases after introducing the CoT module. Compared to models using horizontal bounding box annotations, R_YOLOv5s, CoT_R_YOLOv5s, and the proposed CoT_WRPNet, which use rotated bounding box annotations, have higher parameter counts and computational complexity, adding approximately 0.5 M parameters and 1.5 GFLOPs to the baseline models, while the frame rate decreases slightly. This is mainly because rotated bounding box annotations introduce an angle parameter *β* into the target representation. The model needs to predict the target’s angle while regressing its position and scale, thereby increasing the complexity of the regression task and incurring additional computational overhead. Despite the decrease in frame rate, it still meets the requirements for real-time detection.

Based on the detection accuracy shown in Table 3, each improvement strategy yielded varying degrees of performance improvement. Compared with YOLOv5s, RFPN_YOLOv5s, after introducing the residual feature pyramid, improved its mAP@0.5 by 0.5%; R_YOLOv5s, using rotated bounding box annotation, improved by 3%; and CoT_YOLOv5s, after introducing the CoT module into YOLOv5s, improved by 3.7%. Building on this, WRPNet improved by 3% over RFPN_YOLOv5s and by 0.5% over R_YOLOv5s, indicating that the combination of the weighted residual pyramid structure and rotated bounding box annotation can further improve detection performance. Continuing to introduce the CoT module, CoT_RFPN_YOLOv5s improved by 6.9% compared to RFPN_YOLOv5s, and CoT_R_YOLOv5s improved by 1% compared to R_YOLOv5s. Building on this foundation, the proposed CoT_WRPNet achieves a 0.4% improvement over CoT_RFPN_YOLOv5s and a 3.8% improvement over CoT_R_YOLOv5s, yielding the best detection performance among all models. Overall, the results demonstrate that the proposed CoT_WRPNet effectively improves detection accuracy while maintaining real-time performance.

**Figure 15:**
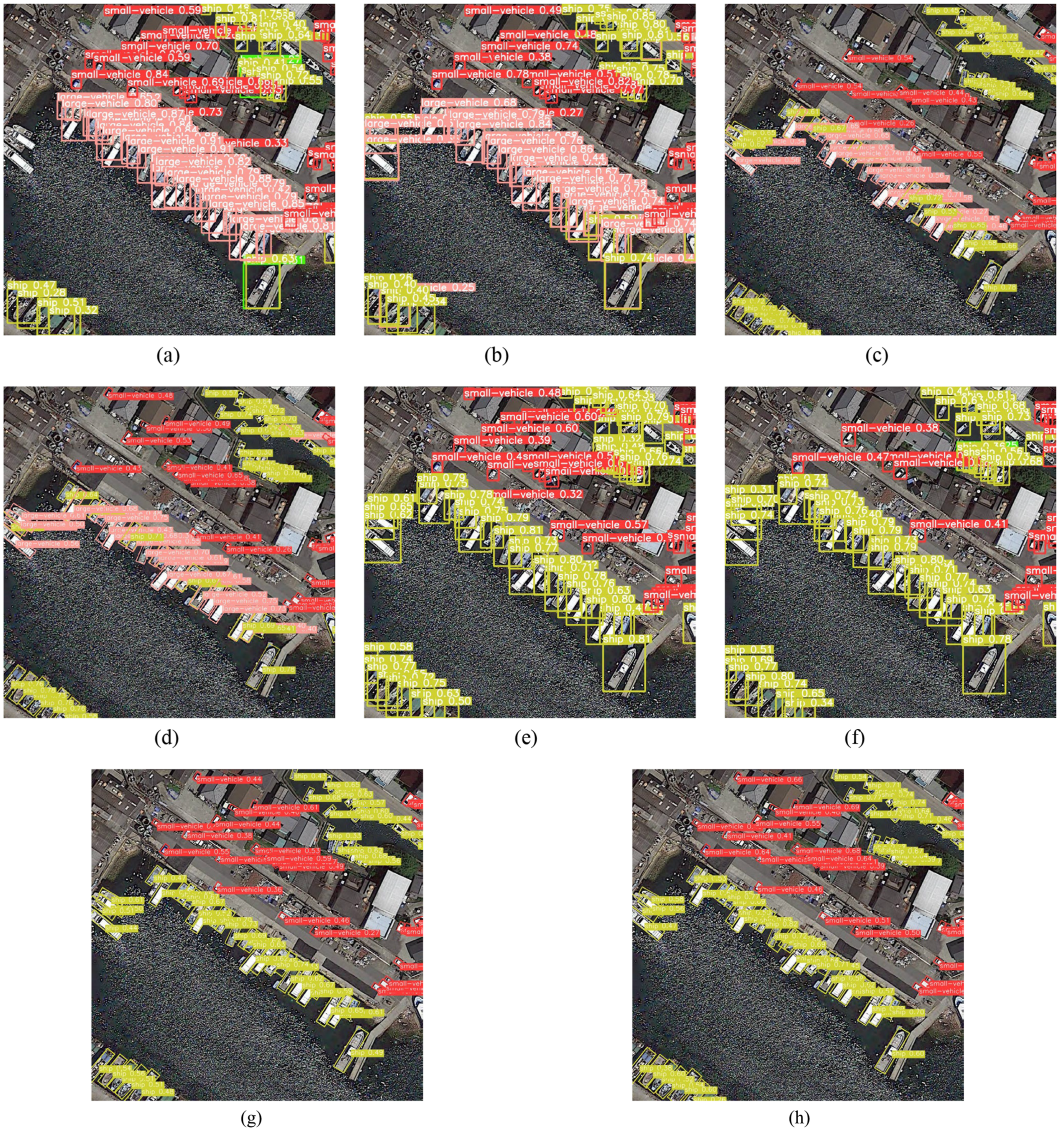
Visual detection results of a scene in the DOTAv1.0 dataset. **(a)** YOLOv5s model; **(b)** RFPN_YOLOv5s model; **(c)** R_YOLOv5s model; **(d)** WRPNet model; **(e)** CoT_YOLOv5s; **(f)** CoT_RFPN_YOLOv5s; **(g)** CoT_R_YOLOv5s; **(h)** CoT_WRPNet (Ours).

Due to space limitations, only the experimental results of some images are visualized. Figure 15 shows the visualization results of eight models in specific scenarios in the DOTAv1.0 dataset.

Figure 15 shows the detection results of different models on the DOTAv1.0 dataset. The rectangles in the figure represent the detected target locations, and different-colored boxes are used to distinguish targets; the numerical value above each box represents the confidence score for that category, and the corresponding category name is labeled next to it. For example, in Figure 15g, the target detected by the yellow box is “ship,” with a confidence score of 0.47. The visualization results show that the DOTAv1.0 dataset has a dense distribution of targets and contains many small-scale targets, which place higher demands on the feature representation capabilities and localization accuracy of detection models. In this complex scenario, some models are prone to missed detections or incomplete detections. In contrast, CoT_WRPNet can detect more valid targets in dense target regions, reducing missed detections and providing more complete results. These results demonstrate that introducing the weighted residual pyramid structure and the contextual Transformer module enhances the model’s target representation capabilities in complex scenes and improves the detection performance of dense small targets, further validating the effectiveness of the proposed method.

#### 4.3.2 Experimental results and analysis of the DOTAv1.5 dataset

This experiment was conducted on the DOTA v1.5 dataset. This section conducted eight experiments. The DOTA v1.5 dataset has 16 categories, with numerous small objects and complex backgrounds. Compared to the DOTA v1.0 dataset, this dataset adds a container crane category, which is less annotated. The entire test set contains 107,333 annotated object instances, of which only 23 are container cranes. This significantly exacerbates the imbalance in how different data samples contribute to the loss. The experimental results are shown in Table 4.

**Table 4:** Ablation results on the DOTAv1.5 dataset.

Method model	YOLOv5s	RFPN_YOLOv5s	R_YOLOv5s	WRPNet	CoT_YOLOv5s	CoT_RFPN_YOLOv5s	CoT_R_YOLOv5s	CoT_WRPNet (Ours)
Baseline (CSPDarkNet53)	√	√	√	√	√	√	√	√
RFPN		√		√		√		√
Rotating Frame			√	√			√	√
CoT					√	√	√	√
Network Layers	213	213	213	213	287	287	303	303
Parameters×10^6^	7.053277	7.053285	7.538737	7.538745	6.344669	6.344677	7.500081	7.500089
GFLOPs	15.9	15.9	17.4	17.4	15.5	15.5	17.4	17.3
FPS	269	267	173	171	50	49	51	48
P(all)	0.804	0.815	0.723	0.761	0.749	0.8	0.694	0.689
R(all)	0.598	0.601	0.627	0.615	0.635	0.622	0.663	0.69
mAP@0.5	0.64	0.645	0.673	0.68	0.669	0.684	0.682	**0.714**

The bold values indicate the best mAP@0.5.

Table 4 presents a performance comparison of the various models. In terms of model complexity, the trend is largely consistent with the results in Table 3 on the DOTAv1.0 dataset. The number of network layers in YOLOv5s, RFPN_YOLOv5s, R_YOLOv5s, and WRPNet remains unchanged, while the number of network layers increases after introducing the CoT module. Compared to models using horizontal bounding boxes, R_YOLOv5s, CoT_R_YOLOv5s, and CoT_WRPNet, which use rotated bounding boxes, show improvements in both parameter count and computational complexity, increasing by approximately 0.5 M parameters and 1.5 GFLOPs compared to the baseline models, while the frame rate decreases slightly. This change primarily concerns the rotation box annotation method. Rotation boxes introduce an angle parameter *β* into the target representation. The model needs to predict the target’s orientation while regressing its position and scale, which makes the regression process more complex and increases parameter and computational overhead. Although the frame rate has decreased, it still meets the basic requirements for real-time detection.

As shown in Table 4, the detection accuracy results on the DOTAv1.5 dataset demonstrate that each improvement strategy improved model performance to varying degrees. Compared to the baseline model YOLOv5s, RFPN_YOLOv5s, which incorporates a residual feature pyramid, improved mAP@0.5 by 0.5%, while R_YOLOv5s, which uses rotated bounding box annotations, improved by 3.3%, indicating that rotating the bounding box significantly improves the accuracy of target localization. CoT_YOLOv5s, which incorporates the CoT module into YOLOv5s, improved by 2.9%, demonstrating that contextual information enhances the model’s understanding of target features, especially in complex contexts, improving detection performance. WRPNet, which combines a weighted residual pyramid with rotated bounding box annotations, achieves a 3.5% improvement over RFPN_YOLOv5s and a 0.7% improvement over R_YOLOv5s. This demonstrates that combining these two improvement strategies can further enhance multi-scale feature representation and target localization capabilities, effectively improving the detection performance for both small and dense targets. Further introducing the CoT module, CoT_RFPN_YOLOv5s achieves a 3.9% improvement over RFPN_YOLOv5s, and CoT_R_YOLOv5s achieves a 0.9% improvement over R_YOLOv5s. This indicates that contextual information can improve detection accuracy across different feature enhancement strategies. Ultimately, the proposed CoT_WRPNet, combining weighted residual pyramids, rotated bounding box annotations, and the CoT module, achieves the highest detection accuracy, representing a 3% improvement over CoT_RFPN_YOLOv5s and a 3.2% improvement over CoT_R_YOLOv5s. Overall, CoT_WRPNet effectively improves detection performance for dense, multi-scale small targets while maintaining real-time capabilities, thereby validating the synergistic effect of various improvement strategies.

Due to space limitations, only the experimental results of some images are visualized. Figure 15 shows the visualization results of eight models in specific scenarios in the DOTAv1.0 dataset.

**Figure 16:**
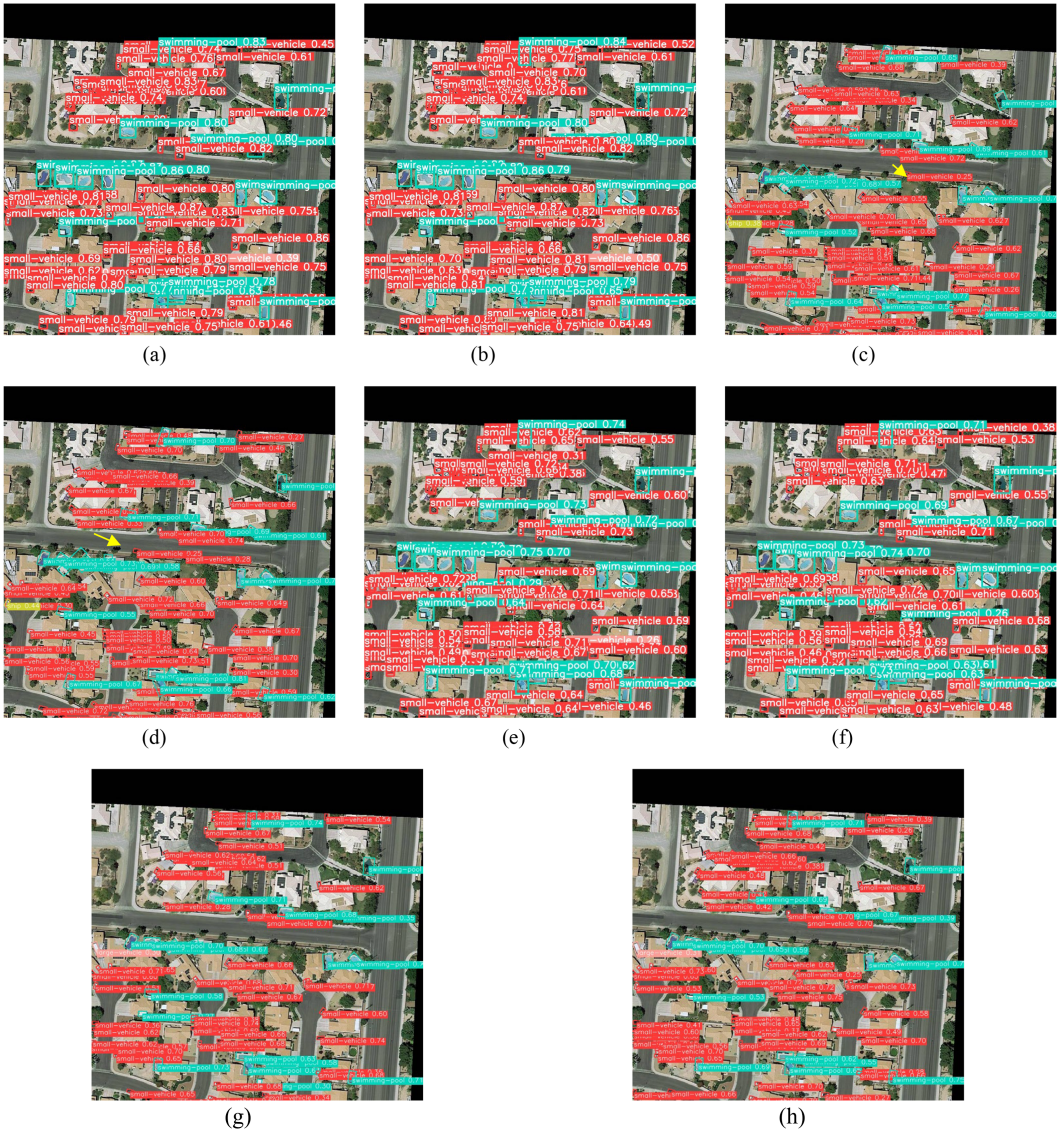
Visual detection results of a scene in the DOTAv1.5 dataset. **(a)** YOLOv5s model; **(b)** RFPN_YOLOv5s model; **(c)** R_YOLOv5s model; **(d)** WRPNet model; **(e)** CoT_YOLOv5s; **(f)** CoT_RFPN_YOLOv5s; **(g)** CoT_R_YOLOv5s; **(h)** CoT_WRPNet (Ours).

In Figure 16, targets are labeled with colored rectangles, with the category name and confidence level displayed on each box. For example, in Figure 16h, the light blue box labeled “swimming pool 0.69” indicates that the target is identified as a swimming pool with a confidence level of 0.69. Comparing Figures 16c,d, one can see that some models exhibit false detections in complex scenes (indicated by yellow arrows), such as misidentifying trees as small vehicles. At the same time, CoT_WRPNet accurately identifies these targets without such errors. This demonstrates that CoT_WRPNet has significant advantages in feature representation and target discrimination. On the one hand, the weighted residual pyramid structure enhances the fusion of multi-scale features, enabling the model to capture target features of different sizes more accurately. On the other hand, the contextual Transformer module leverages information from the surrounding environment to support target judgment, thereby improving the ability to distinguish between adjacent or similarly shaped targets. Results show that CoT_WRPNet achieves higher detection accuracy for dense targets and complex backgrounds, with significantly reduced false-negative and false-positive rates, validating the effectiveness of combining the weighted residual pyramid with contextual information.

### 4.4 Performance and complexity trade-off analysis

Experimental results on the DOTA v1.0 and DOTA v1.5 datasets demonstrate that the proposed CoT_WRPNet model achieves a good balance between detection performance and computational complexity. To further illustrate this characteristic, this section will analyze the model’s performance and complexity.

In the comparative experiments in Section 4.2, CoT_WRPNet achieved an mAP@0.5 of 75.4% on the DOTA v1.0 dataset, which is lower than YOLOv9m’s 78.7% and YOLOv10m’s 79.2%. However, its parameter count is only 0.37 times that of YOLOv9m and 0.49 times that of YOLOv10m, and its GFLOPs are only 0.23 times that of YOLOv9m and 0.29 times that of YOLOv10m. Meanwhile, its FPS is 1.16 times that of YOLOv9m and 1.61 times that of YOLOv10m. On the DOTA v1.5 dataset, CoT_WRPNet achieves an mAP@0.5 of 71.4%, lower than YOLOv9m’s 74.8% and YOLOv10m’s 75.7%. However, its parameter count is only 0.375 times that of YOLOv9m and 0.49 times that of YOLOv10m; its GFLOPs are only 0.23 times those of YOLOv9m and 0.29 times those of YOLOv10m; and its FPS is 1.2 times that of YOLOv9m and 1.37 times that of YOLOv10m. Experimental results show that, although improved accuracy is usually accompanied by higher computational overhead, our model achieves higher inference efficiency while maintaining strong detection performance by significantly reducing the number of parameters and computational cost, thereby striking a reasonable balance between performance and complexity. In comparison with Faster R-CNN and RT-DERT-1, the proposed model demonstrates superior performance in both accuracy and complexity. Furthermore, in comparative experiments with YOLOv8n, YOLOv5s, YOLOv5m, and YOLOx, CoT_WRPNet achieves higher detection accuracy under comparable complexity, further validating the effectiveness of the proposed model in balancing accuracy and complexity.

In the ablation experiments in Section 4.3, on both the DOTA v1.0 and DOTA v1.5 datasets, the introduction of the CoT module, RFPN, and rotated bounding boxes resulted in a stable improvement in mAP@0.5 while maintaining the existing parameter count, GFLOPs, and real-time performance. This indicates that the performance improvement does not entirely depend on the increase in computational complexity.

Therefore, combining the ablation and comparative experiments, it can be seen that our proposed model achieves an effective improvement in detection performance without significantly increasing complexity and while maintaining real-time performance, demonstrating a good balance between accuracy and complexity.

### 4.5 Failed experiments and analysis

During the research process, the team conducted various experiments to more effectively evaluate the impact of different methods on object detection models. Although some experiments failed to improve model performance significantly, these exploratory experiments provided a valuable reference for the team’s further understanding of YOLO-based object detection models. Tables 5, 6 present some failed exploratory experiments on the Dota 1.0 and Dota 1.5 datasets.

**Table 5:** Failed experiments result in the DOTAv1.0 dataset.

Network model	Network layers	Parameters×10^6^	GFLOPs	FPS	P(all)	R(all)	mAP@0.5
YOLOv5s	213	7.05058	15.9	276	0.715	0.673	0.676
RFPN_YOLOv5s	213	7.050588	15.9	284	0.74	0.667	0.681
R_YOLOv5s	213	7.53604	17.4	208	0.764	0.664	0.706
WRPNet	213	7.536048	17.4	207	0.76	0.677	0.711
WRPNet_C2f	201	8.775088	21.1	50	0.735	0.641	0.695
CoT_WRPNet_C2f	214	8.106672	20.6	51	0.703	0.598	0.64

**Table 6:** Failed experiments result in the DOTAv1.5 dataset.

Network model	Network layers	Parameters×10^6^	GFLOPs	FPS	P(all)	R(all)	mAP@0.5
YOLOv5s	213	7.053277	15.9	269	0.804	0.598	0.64
RFPN_YOLOv5s	213	7.053285	15.9	267	0.815	0.601	0.645
R_YOLOv5s	213	7.538737	17.4	173	0.723	0.627	0.673
WRPNet	213	7.538745	17.4	171	0.761	0.615	0.68
WRPNet_C2f	201	8.775088	21.1	50	0.731	0.554	0.605
CoT_WRPNet_C2f	214	8.106672	20.6	51	0.777	0.533	0.602

In Tables 5, 6, the WRPNet_C2f model replaces the C3 module in the WRPNet backbone with the C2f module from YOLOv5; the CoT_WRPNet_C2f model integrates a context transformer module into WRPNet_C2f. Table 4 shows that after replacing the C3 module with the C2f module, the experimental results in both the Dota1.0 and Dota1.5 datasets indicate that, although the number of network layers decreased slightly, GFLOPs increased. At the same time, FPS, P, R, and mAP@0.5 have dropped significantly. Analysis shows that this may be because C2f reduces the multiple bottleneck stacks in the original C3, thereby weakening the target detection model’s ability to express deep features and interact with information. At the same time, because C2f does not use cross-layer connections among convolutional layers, the model’s ability to capture contextual semantic information is limited. In remote sensing image target detection with complex backgrounds, capturing key features is challenging, leading to poor detection performance. After integrating the contextual transformer module into the WRPNet_C2f module, CoT_WRPNet_C2f saw its P, R, and mAP@0.5 decrease by 3.2, 4.3, and 5.5%, respectively, on the dota1.0 dataset. Its R and mAP@0.5 decreased by 2.1 and 0.3%, respectively, on the Dota1.5 dataset. Analysis reveals that while the contextual transformer can, in theory, better account for the relationship between the target and background by combining self-attention with contextual feature fusion to extract target-instance features, in practice, the C2f module may reduce cross-channel connections in the original YOLOv5 model, thereby weakening feature fusion. Furthermore, the contextual transformer relies heavily on semantic features within the context. If the input feature semantics are insufficient, the transformer’s attention mechanism will extract less critical information, potentially affecting recall and reducing model accuracy.

### 4.6 Visualization of disaster remote sensing scene detection

The ablation and comparison experiments described above validate the effectiveness of the CoT_WRPNet model. To further evaluate the model’s generalization ability, this paper applies the model trained on the DATAv1.5 dataset to real disaster remote sensing images. Since the selected real-world scenes are random samples and not precisely labeled, quantitative evaluation is difficult; therefore, visualization results are used for analysis.

**Figure 17:**
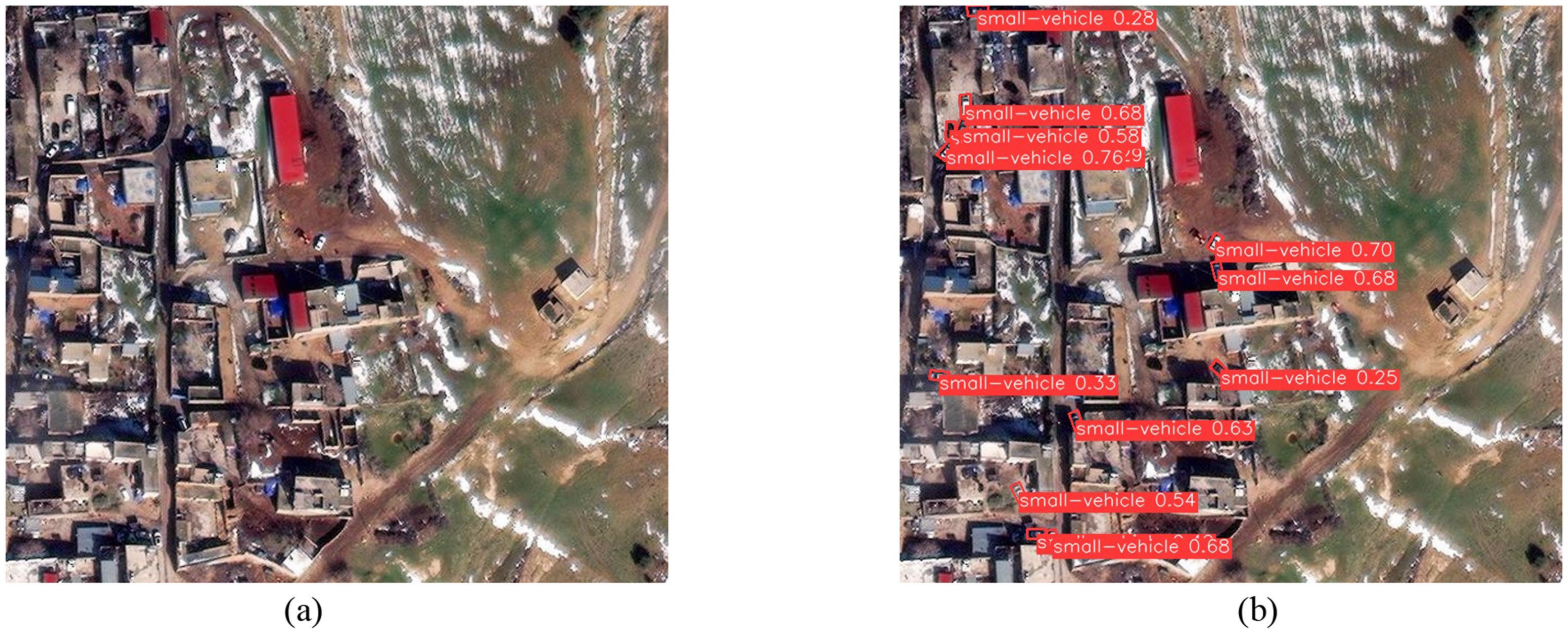
Detection results of an earthquake disaster scene. **(a)** Original image; **(b)** Network detection output.

Three disaster images from different scenarios were randomly selected and directly detected using the CoT_WRPNet model, which was trained on the DATAv1.5 dataset. Figure 17 shows the detection results of the proposed model on any post-earthquake remote sensing image.

Figure 17 is a randomly selected image of the aftermath of an earthquake, showing small vehicles, trees, and buildings. The model proposed in this paper, trained on the DATAv1.5 dataset, can only recognize features for 16 categories, including small vehicles. As shown in Figure 17b, after the new image data is fed into the network, the model can detect the categories and locations of these targets; for example, small vehicles can be effectively identified. In the detection result graph, the target’s position is marked with rectangular boxes of different colors, and the target’s category confidence probability and name are displayed on each box. For example, the red rectangular box of small vehicle 0.68 in figure (b) indicates that the target is a small vehicle with a confidence of 0.68. Since the model in this paper has not learned category features such as tree, it cannot recognize these targets. At the same time, the model also suffers from false positives and missed detections, which the team will investigate further.

**Figure 18:**
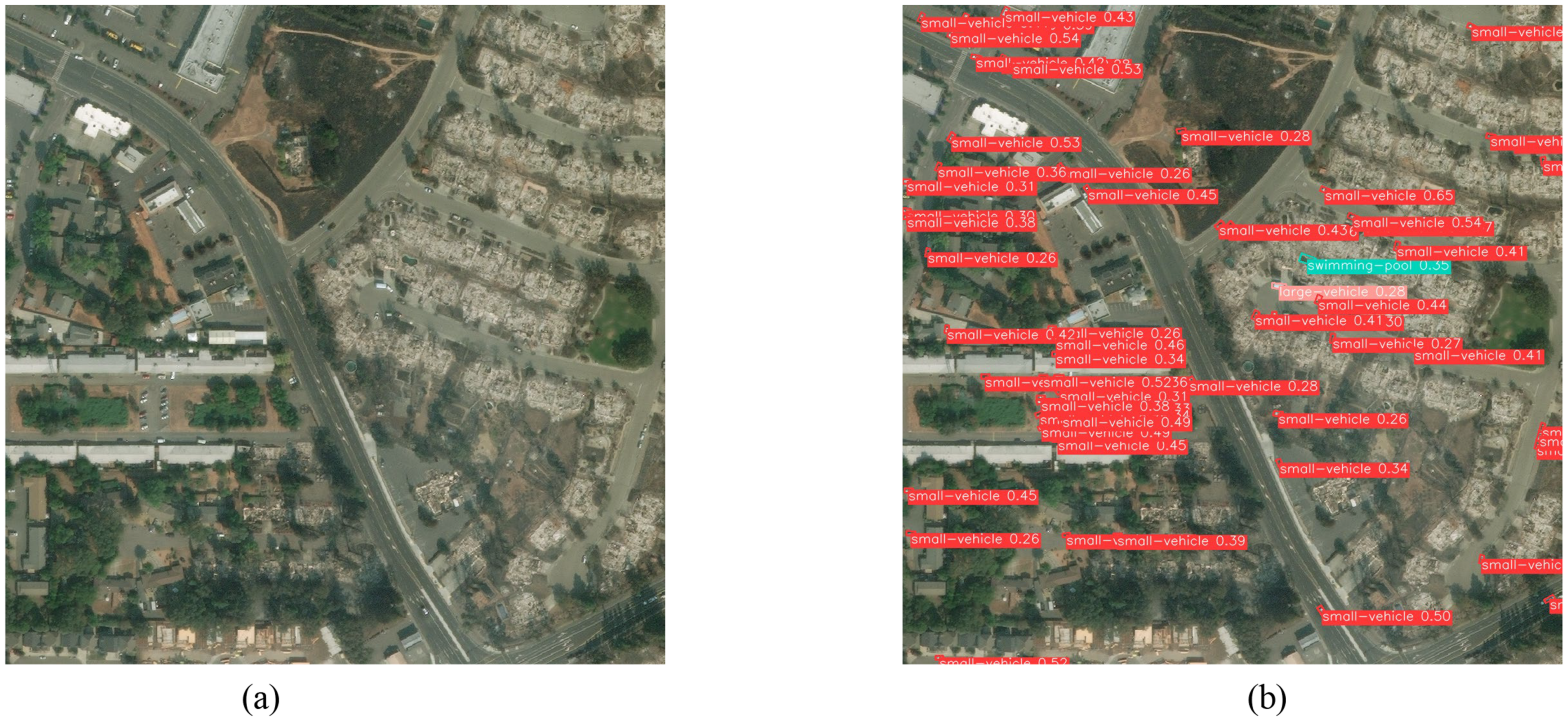
Detection results of the wildfire disaster scene. **(a)** Original image; **(b)** Network detection output.

Figure 18 is a randomly selected remote-sensing image after a wildfire disaster. The image contains scenes, including a small vehicle. The CoT_WRPNet model can only recognize features of 16 categories in the Dota 1.5 dataset, such as small vehicles. As shown in Figure 18b, after the new image data is input into the network, the small vehicle and swimming pool can be effectively identified. In the detection result diagram, the target’s position is marked with rectangular boxes of different colors, and the category confidence probability and the target’s name are displayed on these boxes. For example, the red box of the small vehicle is 0.65 in Figure 18b, which indicates that the confidence that the target is a small vehicle is 0.65. Since the model in this paper has not learned category features such as road, it cannot recognize these target instances, and there are also a small number of missed detections. The team will conduct more in-depth research later.

**Figure 19:**
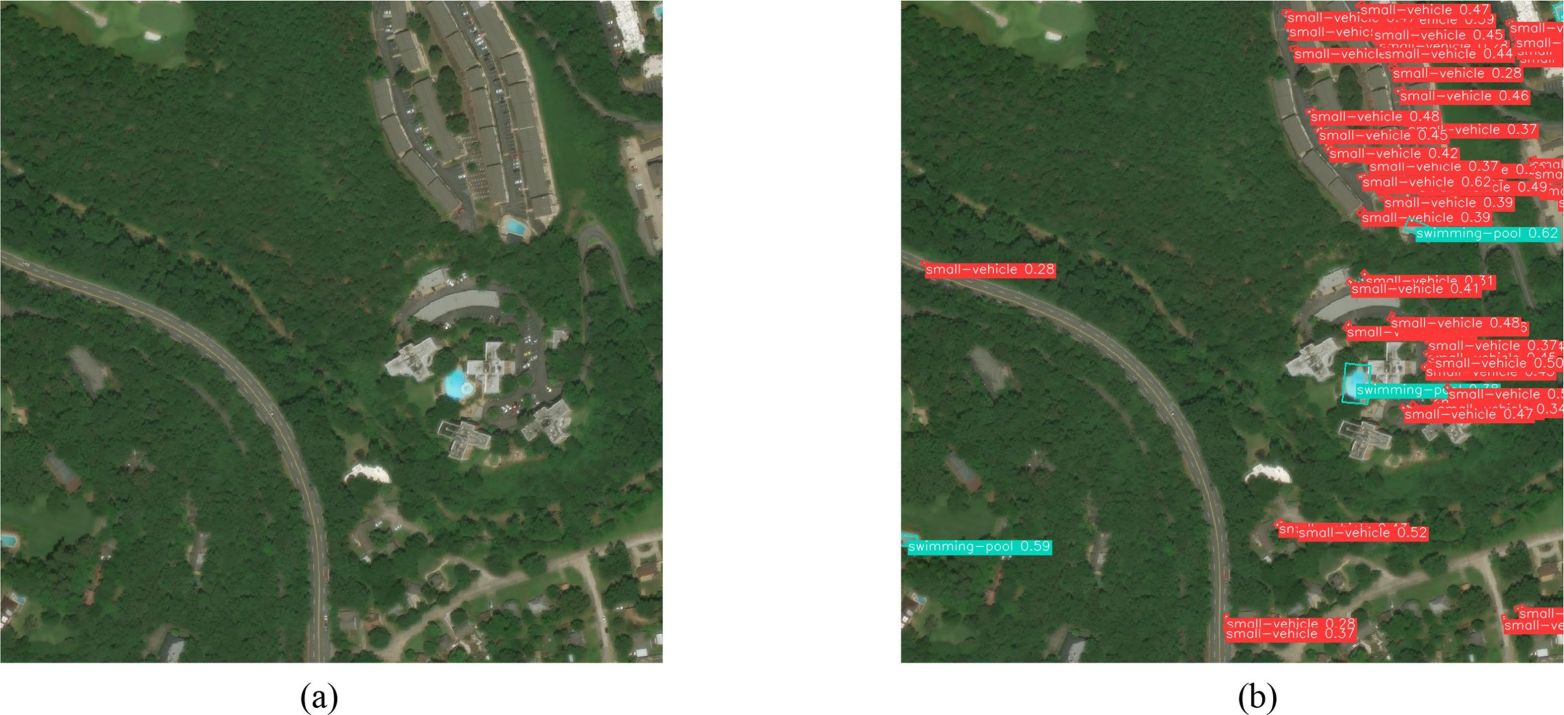
Detection results of the flooding disaster scene. **(a)** Original image; **(b)** Network detection output.

Figure 19 is a randomly selected remote-sensing image after a flooding disaster. The image includes categories such as vegetation, buildings, and small vehicles. As seen in Figure 19b, the model in this paper can effectively detect the category and location information of the small vehicle and swimming pool contained in the image. The target’s location is marked with rectangular boxes of different colors in the figure. The category confidence probability and the target category name are displayed in the rectangular box. For example, the green rectangular box in Figure 19b shows a small vehicle 0.52, indicating the target’s confidence in a small car is 0.52.

The application results of three disaster remote sensing images from various scenarios effectively validated the generalization capability of our model. However, some small objects were missed, and the team will conduct further research in future studies.

## 5 Conclusions and future work

Due to the characteristics of remote sensing images, target instances exhibit large-scale variations, irregular arrangements, and complex backgrounds. Traditional horizontal bounding boxes easily introduce redundant background information, and existing target detection models are insufficient in multi-scale feature fusion and contextual information representation, easily leading to loss imbalance during training. To address these issues, this paper constructs a target detection model, CoT_WRPNet, that combines the Context Transformer and a weighted residual pyramid. Specifically, a weighted residual pyramid module is designed to fuse deep and shallow target-instance features effectively, and a learnable balancing factor is introduced to alleviate the imbalance in contributions across network layers. Simultaneously, a Context Transformer module is introduced into the feature extraction and fusion networks to enhance the model’s ability to represent multi-scale contextual features. Furthermore, rotated bounding boxes are introduced to locate target instances, reducing the influence of redundant background information, and a CIoU-based multi-task loss function is designed to reduce the impact of different target instances on the regression task loss. Ablation and comparative experiments on the DOTA v1.0 and DOTA v1.5 datasets show that the proposed model achieves mAP@0.5 of 0.714 and 0.754, respectively, while maintaining real-time performance, and demonstrates good detection performance compared with multiple methods. The results indicate that the proposed model improves detection accuracy without significantly increasing complexity, achieving a good balance between the two. Furthermore, visualization of detection results across various disaster remote sensing image scenarios demonstrates that the proposed model performs well in complex scenes.

However, the proposed model still incurs overhead in parameter size and computational complexity, and inference efficiency and detection accuracy require further optimization. Future work will consider introducing methods such as model pruning and quantization to reduce computational costs further and improve overall efficiency while maintaining detection performance.

## Data Availability

The raw data supporting the conclusions of this article will be made available by the authors upon reasonable request.
